# Coalescent models characterize sources and demographic history of recent round goby colonization of Great Lakes and inland waters

**DOI:** 10.1111/eva.12779

**Published:** 2019-03-23

**Authors:** Nicholas Sard, John Robinson, Jeannette Kanefsky, Seth Herbst, Kim Scribner

**Affiliations:** ^1^ Department of Fisheries and Wildlife Michigan State University East Lansing Michigan; ^2^ Michigan Department of Natural Resources East Lansing Michigan; ^3^ Department of Integrative Biology Michigan State University East Lansing Michigan; ^4^ Present address: Biology Department SUNY Oswego Oswego New York

**Keywords:** approximate Bayesian computation, aquatic invasive species, coalescence, colonization, demography

## Abstract

The establishment and spread of aquatic invasive species are ecologically and economically harmful and a source of conservation concern internationally. Processes of species invasion have traditionally been inferred from observational data of species presence/absence and relative abundance. However, genetic‐based approaches can provide valuable sources of inference. Restriction site‐associated DNA sequencing was used to identify and genotype single nucleotide polymorphism (SNP) loci for Round Gobies (*Neogobius melanostomus*) (*N* = 440) from 18 sampling locations in the Great Lakes and in three Michigan, USA, drainages (Flint, Au Sable, and Cheboygan River basins). Sampled rivers differed in size, accessibility, and physical characteristics including man‐made dispersal barriers. Population levels of genetic diversity and interpopulation variance in SNP allele frequency were used in coalescence‐based approximate Bayesian computation (ABC) to statistically compare models representing competing hypotheses regarding source population, postcolonization dispersal, and demographic history in the Great Lakes and inland waters. Results indicate different patterns of colonization across the three drainages. In the Flint River, models indicate a strong population bottleneck (<3% of contemporary effective population size) and a single founding event from Saginaw Bay led to the colonization of inland river segments. In the Au Sable River, analyses could not distinguish potential source populations, but supported models indicated multiple introductions from one source population. In the Cheboygan River, supported models indicated that colonization likely proceeded from east (Lake Huron source) to west among inland locales sampled in the system. Despite the recent occupancy of Great Lakes and inland habitats, large numbers of loci analyzed in an ABC framework enable statistically supported identification of source populations and reconstruction of the direction of inland spread and demographic history following establishment. Information from analyses can direct management actions to limit the spread of invasive species from identified sources and most probable vectors into additional inland aquatic habitats.

## 1 INTRODUCTION

The establishment and spread of invasive species have caused devastating trophic cascades (Strayer, 2010) that have reduced the abundance (Gallardo, Clavero, Sánchez, & Vilà, 2016) and diversity (Hejda, Pyšek, & Jarošík, 2009) of native species. Rapid and undesirable shifts in biological community diversity often also result in substantial negative economic costs (Walsh, Carpenter, & Vander Zanden, 2016). Given the deleterious effects associated with biological invasions, conservation agencies have emphasized prevention of human‐assisted (Lockwood, Cassey, & Blackburn, 2005) introductions of invasive species from native sources along with efforts to limit spread of invasive species in habitats that have experienced introductions. These actions are widely believed to be the most cost‐effective control strategies (Mack et al., 2000). However, unambiguous identification of source populations, vectors of dispersal, and demographic changes associated with colonization are often unavailable (Sakai et al., 2001; Lee, 2002). Such information is critical for recently colonized invaders that are increasingly targeted for management actions because this information can be used to prevent future introductions and spread of invasive species.

General information regarding source populations and potential dispersal mechanisms following an invasive species colonizing new habitat can be inferred from presence/absence and relative abundance data collected across a landscape (Jude, Reider, & Smith, 1992; Kot, Lewis, & Driessche, 1996; Pratt, Blust, & Selgeby, 1992). Alternatively, genetic data are widely used to infer founding history and population demographic events (Gaither et al., 2013; Lombaert et al., 2011) and to characterize the magnitude and direction of gene flow among locales (Alp, Keller, Westram, & Robinson, 2012; Estoup, Beaumont, Sennedot, Moritz, & Cornuet, 2004). Model‐based approaches that allow researchers to reconstruct histories of species invasions using demographic models (Estoup & Guillemaud, 2010) are becoming more common because models enable the rigorous evaluation of multiple competing hypotheses concerning the founding sources and demographic histories to inform conservation actions.

Approximate Bayesian computation (ABC) is a generalized, simulation‐based statistical framework that has been widely used for model‐based inference (Beaumont, 2010; Bertorelle, Benazzo, & Mona, 2010; Cornuet et al., 2008; Csilléry, Blum, Gaggiotti, & Francois, 2010; Lintusaari, Gutmann, Dutta, Kaski, & Corander, 2017). ABC has been applied extensively in population genetic studies to reconstruct colonization history (Knowles & Alvarado‐Serrano, 2010; Robinson, Hall, & Wares, 2013; Rougemont & Bernatchez, 2018; Scribner et al., 2017) and the history of species invasions (Ascunce et al., 2011; Benazzo, Ghirotto, Vilaca, & Hoban, 2015; Estoup, Wilson, Sullivan, Cornuet, & Moritz, 2001; Pascual et al., 2007). In an ABC framework, the posterior probabilities of competing hypotheses are approximated on the basis of a set of summary statistics calculated for an empirical observed dataset and simulated datasets generated under each alternative candidate model. Once supported models are identified, ABC can be used to estimate posterior distributions for demographic parameters of interest (e.g., effective size, bottleneck severity; Beaumont, 2010). Finally, simulated datasets are used to assess the power of the ABC approach for model selection, as well as the accuracy and precision of parameter estimates (Bertorelle et al., 2010).

Much of the ABC work involving invasive species has focused on identifying invasion origins over large spatial scales or on inferring colonization history after long periods of time (dozens to hundreds of generations) following founding event(s) (Estoup et al., 2001; Pascual et al., 2007). Because rapid responses to recently detected invaders are important to the success of management actions (Anderson, 2005), agencies would benefit from information about colonization history during the early invasion stages. However, the strength of bottlenecks, as well as the limited time for population divergence in allele frequency due to genetic drift or occurrence of new mutations in different populations, makes inferences based on population genetic data challenging. Importantly, the generation time of the species and the effective population size (which determines the rate of coalescence in the population, Charlesworth, 2009) need to be considered because they affect the efficacy of using population genetic data for demographic inference (Gutenkunst, Hernandez, Williamson, & Bustamante, 2009).

Prior simulation studies and empirical applications have shown that recent events (on a coalescent time scale) pose substantial difficulty for accurate estimation of model parameters (Benazzo et al., 2015; Robinson, Bunnefeld, Hearn, Stone, & Hickerson, 2014; Robinson, Coffman, Hickerson, & Gutenkunst, 2014; Shafer, Gattepaille, Stewart, & Wolf, 2015). However, inferences on short timescales (<20 generations) may be improved by sampling larger numbers of loci (Robinson, Bunnefeld et al., 2014; Shafer et al., 2015) and/or individuals (Keinan & Clark, 2012; Lombaert et al., 2014). Next‐generation sequencing technologies, including massively parallel sequencing of reduced representation genomic libraries (Andrews & Luikart, 2014), have progressed to the point that it is now possible to genotype hundreds of individuals at thousands of loci. Encouragingly, simulation studies suggest that aspects of colonization history can be correctly inferred in as few as 10 generations when using genomic‐scale datasets in an ABC framework (Elleouet & Aitken, 2018). However, there are few applied examples available to evaluate such claims in the literature to date.

The Round Goby, *Neogobius melanostomus *(Pallas, 1814), is a species native to central Eurasia that invaded the Great Lakes in 1990 (Jude et al., 1992), as a result of human‐assisted movements in the ballast water of container ships originating from the Black and Caspian Seas (Brown & Stepien, 2009). Round Gobies were observed in the St. Clair (STC) River in 1990, but by 1993 they had established populations near shipping ports in southern Lake Michigan (LKM) near Chicago, Illinois and central Lake Erie (LKE), near Cleveland, Ohio (Fuller et al., 2018). By 1995, the Round Goby had invaded all five Great Lakes. To date, Round Gobies have colonized much of the Great Lakes shoreline with estimated population sizes in the billions (Johnson, Allen, Corkum, & Lee, 2005). In 1996, the first inland invasion was reported in the Saginaw River Basin in Michigan, and currently, there are numerous examples of inland invasions that resulted from secondary spread from established Great Lakes sources (Fuller et al., 2018). The establishment of Round Goby populations in the Great Lakes and inland waterways within the basin has affected predator–prey dynamics, led to direct competition with native species for resources, and enabled the spread of pathogens (reviewed by Kornis, Mercado‐Silva, & Vander Zanden, 2012).

The rapid colonization of the Great Lakes by Round Gobies and their secondary spread to inland systems may have resulted from multiple Great Lakes source populations and different dispersal mechanisms. First, the secondary spread of Round Gobies may have involved both limited natural dispersal and long‐distance movements in the ballast water of container ships operating within the Great Lakes (Bronnenhuber, Dufour, Higgs, & Heath, 2011; Johansson et al., 2018; LaRue, Ruetz, Stacey, & Thum, 2011). In addition, anglers moving and releasing unused live bait inland may have aided secondary invasions (Drake & Mandrak, 2014; Johnson, Ricciardi, & Carlton, 2001; Leung, Bossenbroek, & Lodge, 2006). However, the specific source(s) of the inland Round Goby invasions, the demographic history associated with founder events, and the mechanisms by which the species colonized fragmented inland systems remains unclear.

In this study, population genomics data and ABC were used to compare models of the Round Goby invasion around the Lower Peninsula of Michigan in Great Lakes waters, as well as within three inland river basins that are fragmented by one or more dams and impoundments. The candidate models included alternative source populations for each inland invasion and several potential colonization routes. Similarities across the three sampled drainages (e.g., in putative source populations) were of particular interest, in that they could indicate shared mechanisms of inland spread. The power of the ABC approach for model selection and the accuracy and precision of parameter estimates were assessed using simulated data. Management actions informed by research outcomes as described in this study for a single target invasive species would have wide‐ranging implications because numerous invasive species have similar life histories and spread dynamics.

## 2 METHODS

### 2.1 Field sampling and library preparations

**Figure 1:**
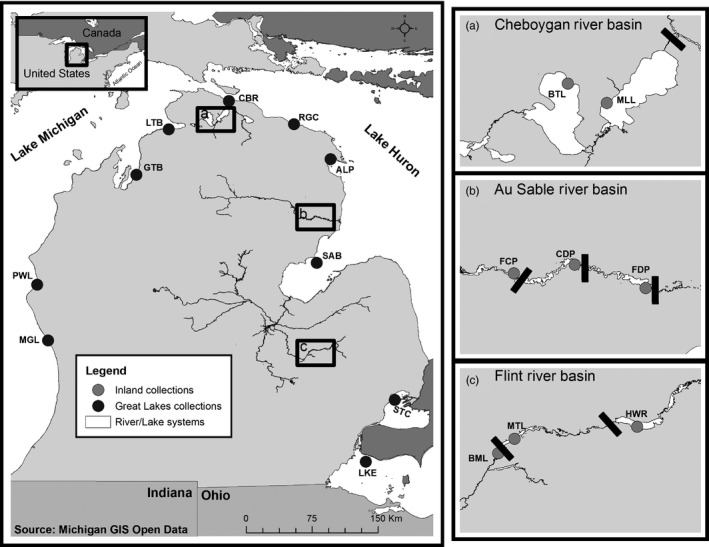
Sampling locations for Round Gobies collected in 2016. Inset maps a–c depict each river basin evaluated. Bold line segments transecting rivers represent impoundments impeding natural dispersal. See Table 1 for sampling information associated with abbreviations

From June to September 2016, Round Gobies were sampled from 18 locations in the Lower Peninsula of Michigan using seines, fishing gear, or backpack electrofishing (Table 1). Collections represent populations from the Great Lakes (e.g., Little Traverse Bay [LTB], Saginaw Bay [SAB], LKE, Lake STC) and populations from three inland river systems (Flint River, Au Sable River, and Cheboygan River systems; Table 1, Figure 1). These three river basins were chosen because they vary in levels of recreational use, distance from populated areas, and number or type of human‐constructed barriers that impede natural upstream movement, all of which have been identified as important factors associated with the secondary spread of aquatic invasive species (Drake & Mandrak, 2010, 2014; Johnson, Olden, & Vander Zanden, 2008). All tissue samples were preserved in 95% ethanol in the field and returned to the lab for DNA extraction.

**Table 1:** Summary information for locations where Round Gobies were collected in 2016

System	Location	Abbreviation	*N*	*H*_e_	pS	*S*
Au Sable River	Foote Dam Pond	FDP	21	0.190	0	1,530
	Cooke Dam Pond	CDP	25	0.189	0	1,617
	Five Channels Pond	FCP	25	0.196	0	1,780
Cheboygan River	Burt Lake	BTL	20	0.195	1	1,577
	Mullett Lake	MLL	25	0.193	1	1,609
Flint River	Below Mott Lake	BML	25	0.137	0	822
	Mott Lake	MTL	25	0.137	0	820
	Holloway Reservoir	HWR	25	0.116	0	762
Potential sources	Alpena	ALP	25	0.191	1	1,754
	Cheboygan River	CBR	25	0.200	0	1,847
	Grand Traverse Bay	GTB	25	0.174	1	1,469
	Lake Erie	LKE	25	0.202	3	1,913
	Lake St. Clair	STC	25	0.206	3	1,807
	Little Traverse Bay	LTB	25	0.193	3	1,695
	Muskegon River	MGL	24	0.190	4	1,545
	Pentwater Lake	PWL	25	0.181	2	1,484
	Roger City	RGC	25	0.202	8	1,849
	Saginaw Bay	SAB	25	0.199	1	1,728

Note

Abbreviated names, the number of individuals sampled per location (*N*), expected heterozygosity (H_e_), the number of private sites (pS), as well as the number of segregating sites (S) are provided for each location.

DNA libraries were prepared using the “BestRAD” protocol described by Ali et al. (2016). Libraries were sequenced with paired‐end 150‐base pair (bp) reads in two Illumina HiSeq 2500 (v4 flow cell) lanes. Associated data were processed using STACKS v. 1.44 (Catchen, Amores, Hohenlohe, Cresko, & Postlethwait, 2011; Catchen, Hohenlohe, Bassham, Amores, & Cresko, 2013). Loci were included in the final VCF file if they were successfully genotyped in 80% of all individuals within a population at a depth of ≥9× coverage and were genotyped in at least 15 of the 18 populations sampled. Detailed sequencing and bioinformatics methods are included in Supporting Information Appendix S1.

### 2.2 ABC model development

We defined a series of plausible models representing a series colonization of events in the Great Lakes in nearshore habitats around Michigan to characterize relationships among Round Goby populations in the Great Lakes. Specifically, based on USGS observation data (Fuller et al., 2018), ports near Chicago in LKM (1993) and the STC River (1990) were colonized within 3 years of each other, and Round Gobies have since slowly colonized Michigan shores of Lakes Michigan and Huron. At some point, these two invasion fronts met, yet there are no published studies that statistically compare models that differ in the location of the juncture between Lake Huron and LKM invasion fronts. Specifically, analyses have not been conducted to ascertain whether Round Goby populations in LTB, or in the lower Cheboygan River, were colonized as a result of range expansions from the original introduction in the STC River, or as a result of expansion from the early colonization of LKM harbors. We used the ABC framework to quantitatively evaluate four hypotheses that differed in the location of the juncture between populations derived from population expansion from Lake STC through Lake Huron and expansion through LKM (Supporting Information Appendix S1, Table A1.1). We delineate populations geographically given the large distances separating all sampled populations in the Great Lakes (Figure 1) and the man‐made barriers that separate the inland segments of sampled river drainages (e.g., Holloway Reservoir, Mott Lake, etc.; Figure 1). While downstream connectivity of individual segments is likely, the presence of dams in the system prevents natural dispersal upstream. Furthermore, as our goal is to provide useful information to managers, we maintained the natural geographic organization of individuals into populations, rather than pooling to reduce complexity of population tree topologies projected under alternative hypotheses of invasion spread (gene flow). Pooling sampling locales within drainages would limit plausible colonization and dispersion scenarios to be explored in our models and the efficacy and interpretability of parameter estimates from our analysis.

We tested multiple competing hypotheses to explain how Round Gobies colonized each of the three sampled drainages (Flint, Au Sable, Cheboygan Rivers) in an ABC framework to assess whether consistent patterns among secondary invasions from the Great Lakes to inland systems could be identified (i.e., inland locales shared the same source, exhibited similar directionality [upstream vs. downstream] following colonization, exhibited similar founding effective sizes, etc.). Models shared a common underlying topology (i.e., population tree, see Supporting Information Appendix S1, Figure A1.1), but varied in the source population associated with each inland invasion. For each inland system, we also considered models that allowed separate introductions into multiple inland populations from a shared source (as in Benazzo et al., 2015). Given the spatial separation of the sampled systems (Figure 1), both alternative and shared source populations were considered in candidate models. Models were informed by data available in the USGS Nonindigenous Aquatic Species database (Fuller et al., 2018). The number of models differed between systems (13 Flint models; 17 Au Sable models; 21 Cheboygan models; Supporting Information Appendix S1, Tables A1.2–A1.4). Given the scale of the analyses conducted, we provide model development details in Supporting Information Appendix S1.

### 2.3 Simulations and statistical analysis

We used the STACKS parameters noted above to set minimum sample sizes from each population (80% of the number of sampled individuals) to allow assembly of a complete genotypic matrix (i.e., no missing data) for each population to be used in analyses. Briefly, for each SNP locus, *n_i_*nonmissing individual genotypes (with *n* set to 80% of the per‐population sample size) were randomly selected without replacement from the initial dataset for each of the *i *sampled populations. This practice preserved associations between variants at each locus within individuals, but breaks associations between SNP loci within individuals by randomly sampling genotypes for the *n_i_*individuals included in the reduced dataset from each population. This process was conducted to improve computational efficiency because preliminary simulations indicated that replicating the pattern of missing data in the observed dataset was time‐intensive (~10× increase in time). Importantly, the above procedure does not affect the statistics considered in the ABC analysis below, as they are based on numbers of variable SNPs and the frequencies of variants at these loci. No linkage‐based statistics were included, as patterns of linkage among loci in the reduced dataset would not match those of the full data.

A central component to ABC inferences is the set of summary statistics used in the analysis. Therefore, it was essential to maximize accuracy and precision of summary statistics estimated (see below), especially under the challenging colonization scenarios investigated here. For the nearshore Great Lakes and each inland system, we used different numbers of SNP loci (range: 2,901–3,699) to maximize genotypic information among locales of interest (at the specified minimum 9× coverage and 80% population genotyping levels) in order to maximize the accuracy and precision of summary statistic estimates used in each ABC analysis. The number of loci likely varies across analyses, in part, because of the stochastic nature of reduced representation genotyping procedures.

Summary measures of intra‐ and intersample genetic diversity were used in an ABC framework to quantitatively compare alternative hypotheses of Round Goby colonization along nearshore Great Lakes habitats including LKM and Lake Huron, and colonization of three sampled systems (Flint River, Au Sable River, and Cheboygan River). We calculated summary statistics that include information associated with genetic diversity of populations, as well as the divergence in SNP allele frequency among populations. Diversity measures included the number of polymorphic loci in each population (and the mean and standard deviation of this number, across populations), the number of private polymorphic sites, hereafter private sites (pS), in each population (and the mean, standard deviation, and total number of private sites based on within population estimates, i.e., one statistic each), and the expected heterozygosity (*H*_e_) at SNP loci in each population (and across all populations). Statistics related to genetic divergence included pairwise (and total) *F*_st_ (using a simple, but computationally efficient estimate based on heterozygosity, *G*_ST_; Nei, 1975), the number of SNP loci that fall along the *x* and *y*‐axes of the joint site frequency spectrum (jSFS) for each pairwise population comparison (i.e., the number of private sites in each pairwise jSFS), and the mean frequency of private (minor allele) sites in each pairwise jSFS. The additional paired statistics provide information related to the directionality associated with gene flow (Slatkin, 1985). Given the paired nature of several of the above measures of divergence, the total number of summary statistics varied with the number of simulated populations considered for the four ABC analyses conducted here (see Supporting Information Appendix S1). All statistics were calculated in the R statistical computing environment (R Core Team, 2016), using a combination of functions from the “strataG” R package (Archer, Adams, & Schneiders, 2017) and custom scripts (available on GitHub: nicksard/2019_Goby_ABC_scripts).

Coalescent simulations of each of the candidate models were conducted in fastsimcoal v. 2.5 (Excoffier, Dupanloup, Huerta‐Sanchez, Sousa, & Foll, 2013) using modified wrapper functions from the “strataG” package (Archer et al., 2017) in the R statistical computing environment (R Core Team, 2016). Functions available in “strataG” and custom scripts (see above) were used to calculate summary statistics for each simulated replicate. For each sampled inland river system and associated competing colonization scenario, one million replicates of each candidate model were simulated for the purposes of ABC‐based model selection and parameter estimation (Bertorelle et al., 2010). All simulations were conducted using computational resources provided by Michigan State University's Institute for Cyber‐Enabled Research.

Approximate Bayesian computation analyses were conducted using the R packages “abc” (Csilléry, François, & Blum, 2012) and “abcrf” (Marin, Raynal, Pudlo, Robert, & Estoup, 2017). Model selection analyses were carried out using neural network (Blum & Francois, 2010) and random forest (Pudlo et al., 2015) methods. Neural networks included five hidden layers and models in the Flint, Au Sable, and Cheboygan analyses were compared at tolerances of 0.001 and 0.005, whereas models tested for the Lower Peninsula analysis were compared at tolerances of 0.01 and 0.005, due to the smaller number of total simulations considered for this analysis. Each random forest analysis used 1,000 trees to predict the most likely model. Models receiving substantial support from these analyses were subsequently used for parameter estimation. Estimated model parameters included contemporary effective size of inland populations, bottleneck severity parameters, and migration rates among locales (see Supporting Information Appendix S1 for more details), again using neural networks (Blum & Francois, 2010) to estimate posterior distributions for model parameters at tolerances of 0.01 and 0.05.

### 2.4 Evaluating the quality of inferences

Pseudo‐observed datasets (PODS) were used to assess the power of our ABC approach for model selection and the accuracy and precision of parameter estimates (Bertorelle et al., 2010). Individual simulations from the reference table (consisting of summary statistics for each of the 1 million replicates per model tested, and the randomly drawn parameter values used to generate each dataset) were used as PODS in leave‐one‐out cross‐validations to test the ability of the ABC analysis to correctly assign known datasets to the model under which they were simulated (Csilléry et al., 2012; Robinson, Bunnefeld et al., 2014; Robinson et al., 2013; Shafer et al., 2015). One hundred cross‐validation replicates per model were conducted for each of the simulated models. These analyses allowed the estimation of misclassification rates for each candidate model (similar to type I and type II error rates; Bertorelle et al., 2010, Lombaert et al., 2014). For the random forest model selection analysis, we recorded the “out‐of‐bag” error rates (proportion of datasets misassigned to alternative models) returned during the construction of the random forest classifier (Pudlo et al., 2015). Due to the number of models considered and the number of populations included in each model, cross‐validations to assess model identifiability in the Cheboygan River system were based on a smaller reference table including 100,000 simulations per model (rather than the full one million simulated) and a single tolerance (0.01, corresponding to 21,000 accepted simulations) to reduce the computational burden associated with this analysis. We expect that this reduced dataset provides a conservative estimate of the ability of ABC to distinguish the candidate models for this system (i.e., we expect either no difference or improvements in model identifiability with the larger reference table used to analyze empirical data).

To evaluate the accuracy of parameter estimates, we conducted an additional 100 leave‐one‐out cross‐validations for each model receiving substantial posterior support in upstream model selection analyses at the lowest tolerance evaluated. These cross‐validations were used to calculate prediction errors for each model parameter using simulated (known) parameter values and their respective point estimates (medians of posterior distributions). Finally, to evaluate the fit of the best models and parameter values for each system (as chosen via ABC), we conducted 1,000 posterior predictive simulations using parameter values randomly drawn from the posterior distributions. Summary statistics from these posterior predictive simulations were compared to those calculated from the observed data, and posterior predictive *p*‐values (the probability that simulated summary statistics are more extreme than observed statistics; Bertorelle et al., 2010) for each summary statistic were calculated.

## 3 RESULTS

### 3.1 Summary of population diversity measures

Initially, we used 2,312 SNP loci genotyped in at least 80% of individuals in all 18 Round Goby populations to estimate and qualitatively compare measures of genetic diversity within and among Great Lakes and inland locations. Among Great Lakes sampling locations, expected heterozygosity at SNP loci was 0.194 ± 0.010 (mean ± *SD*), which was comparable to samples from inland locations in the Au Sable River (0.192 ± 0.004) and Cheboygan River (0.194 ± 0.001) basins. Sample collections in the Flint River basin had lower expected heterozygosity (0.130 ± 0.012) (Table 1), suggesting a bottleneck at the time of founding. Across all sample collections, observed numbers of private sites were low in inland locations, as expected given the recent invasion history by Round Gobies into inland waters (<15 generations). No private sites were observed in most (six of eight) sample collections within the Flint, Au Sable, or Cheboygan River basins.

**Figure 2:**
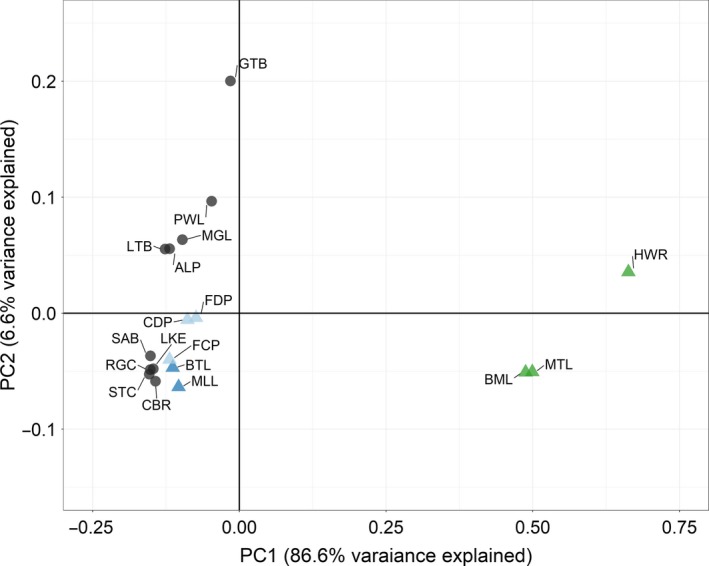
Principle coordinate analysis visually reflecting pairwise *F*_st_ differences among Round Goby sample collections. See Table 1 for abbreviations. Black circles represent potential Great Lakes sources. Triangles represent inland collections, with each basin represented by a unique color—Flint River, green; Au Sable River, light blue; Cheboygan River, dark blue. All points were made semitransparent

We used *F*_st_ as one measure of connectivity or gene flow among populations to identify putative Great Lakes source populations associated with secondary dispersal from original Great Lakes founding populations (Supporting Information Appendix S2, Figure A2.1). Pairwise *F*_st_ estimates among locations within the Flint River (0.054 ± 0.050), Au Sable River (0.036 ± 0.021), and Cheboygan River (0.003) basins were lower relative to pairwise comparisons between inland and Great Lakes locations (Supporting Information Appendix S2, Figure A2.1). Pairwise comparisons between Flint River locations and Great Lakes locations were characterized by the highest *F*_st _estimates (0.200 ± 0.032) observed in the study. Principal coordinate analyses provide a visual representation of population interrelationships based on pairwise *F*_st_ (Figure 2), indicating that each of three collections from the Flint River was similar in allele frequency, but highly differentiated from all other sampling locations. Most Great Lakes collections clustered together. Notably, Cheboygan River, SAB, and STC River samples cluster with inland collections from the Au Sable and Cheboygan Rivers suggesting shared histories. For brevity, we report only *F*_st_ pairwise comparisons; however, information from pairwise jSFS was also used in the ABC analyses below (see Section 2 for more information).

### 3.2 Reconstruction of secondary dispersal

**Figure 3:**
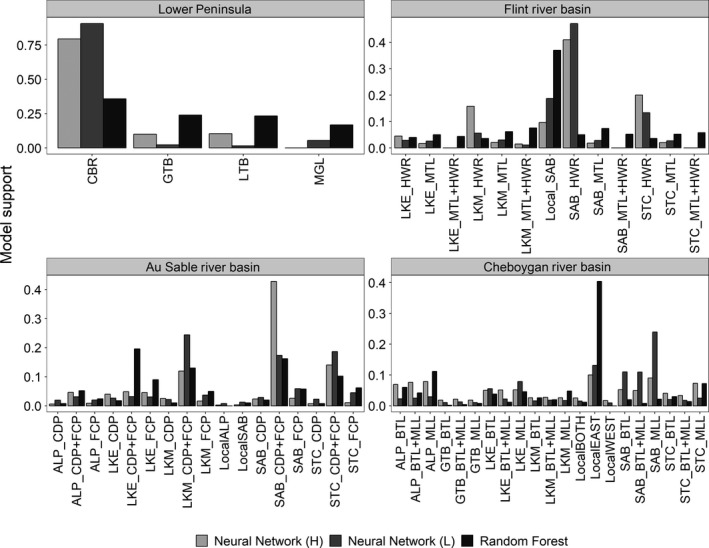
Model support for each hypothesis tested within each approximate Bayesian computation analysis. Model support values for neural network at reported at high and low tolerances. High and low tolerances for the Flint River, Au Sable River, and Cheboygan River analyses were 0.005 and 0.001, respectively, whereas they were 0.01 and 0.005 for the Lower Peninsula analysis. Random forest model support values represent the proportion of votes for a given model during the decision tree process. See Table 1 for location abbreviations and Tables A1.1–A1.4 (Supporting Information Appendix S1) for descriptions of each model tested. Abbreviated hypothesis names represent the source of the introduction (name to the left of the underscore), and the first location(s) that were founded with the system (to the right of the underscore). If multiple introductions occurred, they are indicated with a “+” (e.g., MTL + HWR represents introductions into both Mott Lake and Holloway Reservoir at the same time)

Our ABC analyses of the colonization history of nearshore Great Lakes waters around Michigan's Lower Peninsula indicated that Round Goby populations from LKM, including LTB, Grand Traverse Bay, and Muskegon River (Figure 1), were colonized from a LKM source, whereas the Cheboygan River population was derived from populations in Lake Huron (Table 2). This model was supported based on random forest (probability [Pr] = 0.87) and neural network model selection criteria with tolerances of 0.01 (Pr = 0.79) and 0.005 (Pr = 0.91; Figure 3).

**Table 2:** Summary of major findings from each of the four ABC analyses conducted

ABC analysis	Model(s) supported	Source(s)	Invasion route(s)	Major conclusions
Lower Peninsula	CBR	LKM and Lake STC	Ballast water and natural dispersal from shipping ports	Sample collections in Lake Huron were derived from Lake STC, whereas those collected in LKM were derived from an unsampled source
Flint River	LocalSAB and SAB_HWR	SAB	Single introduction, stepping‐stone colonization	Round Gobies that founded the Flint River came from SAB and colonized the system in a stepping‐stone manner
Au Sable River	Multiple	Unknown	Multiple introductions, one into FCP and the other in Cook Dam Pond	The source of the invasion was not identified, but there is support that the Au Sable River was colonized as the result of multiple introductions
Cheboygan River	LocalEast and SAB_MLL	SAB or mouth of the Cheboygan River	Single Introduction, colonization occurred East to West (Mullet to Burt)	The Cheboygan system was colonized either by long‐distance movement from Round Gobies in SAB or by dispersal through the lock on the Cheboygan River. Evidence suggests that Mullet Lake was founded first and Round Gobies then spread to Burt Lake

Note

ABC: approximate Bayesian computation; CBR: Cheboygan River; FCP: Five Channels Pond; HWR: Holloway Reservoir; LKM: Lake Michigan; MLL: Mullett Lake; SAB: Saginaw Bay; STC: St. Clair.

Approximate Bayesian computation analyses of three inland invasions into the Flint, Au Sable, and Cheboygan River systems indicated support for particular inland invasion routes (Table 2). In the Flint River, model selection suggested that the source of the inland invasion was likely SAB. Both the human‐assisted upstream movement (LocalSAB; random forest, Pr = 0.55) and SAB introduction into Holloway Reservoir (SAB_HWR) models were supported (Table 2, Figure 3). The SAB_HWR model was the most supported model based on neural network model selection (tol. = 0.005, Pr = 0.41). Bayes factors measuring support in favor of the SAB_HWR model over the next best models (MIC_HWR and STC_HWR) were <3 at both tolerances, indicating a lack of strong support for SAB_HWR over alternative models based neural network model selection (Jeffreys, 1961; Figure 3).

Multiple introductions were supported for the Au Sable River system, but models representing introduction from alternative sources were difficult to distinguish. Neural network model selection at either a tolerance of 0.001 or 0.005 indicated multiple introductions into Five Channels Pond (FCP) and Cooke Dam Pond (CDP), which are different impounded regions of the river (Figure 3). Great Lakes sources with the highest support included SAB, Lake STC, and an unsampled LKM population. The same colonization process (multiple introductions from the same source) was also favored using random forest model selection, but from a LKE source (Pr = 0.83).

Analyses of Round Goby colonization of the Cheboygan river system indicated east (Mullett Lake) to west (Burt Lake) colonization within the system (Table 2). Models where the source of invasion was from Saginaw Bay (SAB_MLL) and a model that included colonization through a semipermeable lock at the mouth of the Cheboygan River (LocalEast) both received support. The LocalEast model was the most supported model (Pr = 0.53) based on the random forest model selection. This model represents colonization of the Cheboygan system via movements upstream through the Cheboygan Lock, which connects the Cheboygan River and Mullett Lake to Lake Huron, which is similar to the Local_SAB in the Flint River analysis (Figure 1).

### 3.3 Establishing parameter estimates

**Figure 4:**
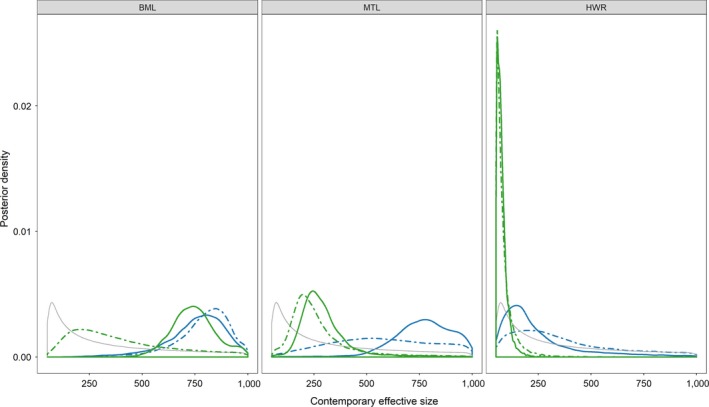
Prior (gray lines) and posterior probability densities for contemporary effective population size estimated in the Flint analysis. Dotted and solid lines represent posterior estimates at 0.05 and 0.01 tolerances for each supported model. LocalSAB (blue) and SAB_HWR (green) models are represented. See Table A1.2 (Supporting Information Appendix S1) for description of the models

An additional component of the ABC analyses involved estimation of parameters including contemporary *N*_e_, migration among sampling locations, and the strength of founding bottlenecks (Supporting Information Appendix S2: Figures A2.2–A2.5). Parameter estimation was conducted for all models receiving support in each ABC model selection analysis. In all four ABC analyses, we found evidence that the mean *N*_e _of Round Goby populations in putative Great Lakes sources was large (>500) among populations. Within the inland locations in the Flint, Au Sable, and Cheboygan River basins, we found that power to estimate contemporary *N*_e_ varied. In the Au Sable River, posterior distributions of contemporary *N*_e _did not deviate substantially from prior distributions. In contrast, posterior distributions for parameters of both supported models for the Flint River (LocalSAB and SAB_HWR) suggest that contemporary *N*_e_ for both the impounded population below Mott Lake and Mott Lake is moderately large (median *N*_e_ > 200), but that *N*_e_ for Holloway Reservoir was substantially smaller (Figure 4, Supporting Information Appendix S2: Figure A2.3). The median *N*_e_ estimate for Holloway Reservoir based on the SAB_HWR model was 69 (51–136, 95% HDI), which was 2.7 times smaller than the LocalSAB estimate (Median: 184, 95% HDI: 61–777). Similarly, in the Cheboygan River system, we found that posterior distributions for contemporary *N*_e_ estimated under SAB_MLL and LocalEast models were consistent (Supporting Information Appendix S2: Figure A2.5). However, point estimates of *N*_e_ in Burt Lake were larger under the LocalEast (median *N*_e_ = 583) model than under the SAB_MLL model (median *N*_e_ = 266).

We lacked the power to estimate parameters associated with migration rates among populations in all inland systems. However, we did observe parameter posterior distributions that differed from prior distributions for bottleneck severity parameters (proportional reduction from contemporary *N*_e_). Initial Round Goby movements within the Great Lakes appear to have been accompanied by relatively minor reductions in *N*_e_. We estimate that *N*_e_ for Round Gobies presumed to have been established via ballast water assisted movement within the Great Lakes were 15% (SF) that of contemporary *N*_e _(Supporting Information Appendix S2: Figure A2.2). Posterior distributions for the severity of bottlenecks associated with natural colonization events following initial colonization indicate that initial founding size for Round Goby dispersing naturally from shipping ports was 9% to 10% (95% HDI, Supporting Information Appendix S2: Figure A2.2) that of contemporary *N*_e_ per population.

Within inland systems, estimated posterior distributions of bottleneck severity parameters indicated that Round Goby populations experienced stronger bottlenecks during the invasion of the inland systems surveyed. In analyses of the Flint River colonization, both the SAB_HWR and the LocalSAB models estimate that strong bottlenecks occurred at the timing of founding at each location surveyed (i.e., River Founding—RF). Estimates of bottleneck severity indicate populations were colonized by founder population 1%–4% (95% HDI) that of current *N*_e_ (Supporting Information Appendix S2: Figure A2.3). Bottleneck severity parameters associated with founding river (RF) populations indicated a weaker bottleneck during colonization of inland portions of the Au Sable and Cheboygan Rivers (median posterior density ~7%, Supporting Information Appendix S2: Figure A2.4 and A2.5) than estimated for the Flint River system (median posterior density <3%, Supporting Information Appendix S2: Figure A2.3).

### 3.4 Cross‐validation and estimation of model fit

**Figure 5:**
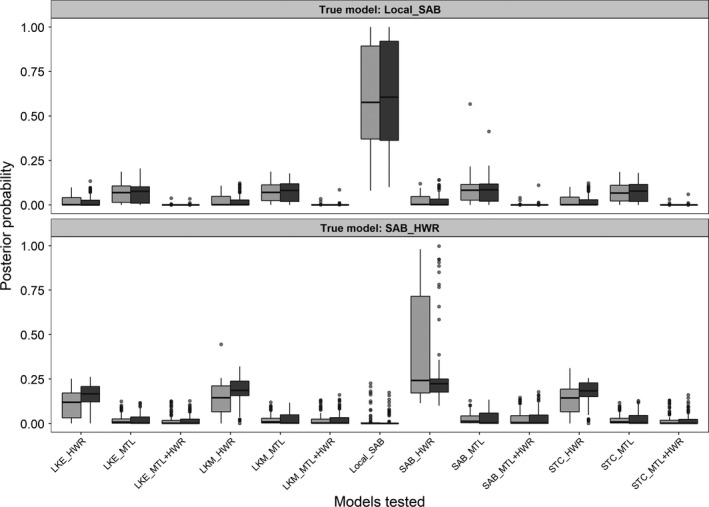
Boxplots depicting 100 leave‐one‐out cross‐validations for neural network analysis at tolerances 0.005 (Light gray) and 0.001 (dark gray) for the two supported models in the Flint River analysis. Abbreviated hypothesis names represent the source of the introduction (name to the left of the underscore), and the first location(s) that were founded with the system (to the right of the underscore). If multiple introductions occurred, they are indicated with a “+” (e.g., MTL + HWR represents introductions into both Mott Lake and Holloway Reservoir at the same time). See Table 1 for location abbreviations and Table A1.2 (Supporting Information Appendix S1) for descriptions of each model tested

For each of the four ABC analyses, we used simulated data for each model to evaluate the power of the neural network and random forest approaches for model selection. In all ABC analyses, we found that routes of invasion (e.g., upstream vs. downstream, single or multiple introductions) within a system could be distinguished, but that it was more difficult to identify specific sources (Supporting Information Appendix S2: Figures A2.6–A2.9, Tables A2.1–A2.4). Figure 5 provides an example of cross‐validation simulations that demonstrate that models representing the same colonization process, but from different sources were more difficult to distinguish in neural network analyses. We observed similar model identifiability issues for random forest analyses as well. For instance, in Au Sable cross‐validations, we observed higher misclassification rates among models that shared an invasion route but differed in the source population responsible for inland colonization (Supporting Information Appendix S2: Table A2.3). As an example, 90% (0.227) of the total misclassification rate (0.253) in the ALP_FCP cross‐validation simulations could be attributed to other models representing the same process (Introduction into FCP, Five Channels Pond, first), but from different sources (i.e., LKE, LKM, SAB, or Lake STC). However, the Great Lakes nearshore ABC models were confidently distinguished from each other using either neural network (i.e., 99% correct model identification) or random forests model selection criteria.

Cross‐validation analyses indicate that posterior distributions provided informative estimates for some parameters (Supporting Information Appendix S2: Figure A2.10–A2.13). Importantly, prediction errors were smaller for bottleneck severity parameters associated with founding a river (RF) within an inland system (range: 0.15–0.38) compared to founding a new port in the Great Lakes via movement associated with the shipping trade (SF, range: 0.34–0.71). Meanwhile, prediction error associated with migration parameters were high (>0.98), indicating that reliable estimates of migration rates could not be obtained in any of the four ABC analyses. In contrast, prediction errors associated with contemporary *N*_e _of inland sampling locations were relatively low across all four ABC analyses (range: 0.18–0.57).

Posterior predictive simulations were used to assess how well the most supported models, and associated parameter estimates, fit observed summary statistics from each dataset. In all four ABC analyses, we found that simulated models produced distributions of summary statistics that overlapped with observed data in a majority of cases, indicating fit of the parameterized model to the data. Observed values of the numerous pairwise statistics (i.e., *G_ST_*, number and frequencies of pairwise private alleles) were adequately captured by the parameterized model. In contrast, observed numbers of several population‐specific statistics (segregating sites, expected heterozygosity, and numbers of private sites per population), which comprise a small fraction of the total statistics used in ABC analyses, often fell outside of the distribution from posterior predictive simulations. In general, discrepancies indicate higher levels of diversity in the observed data (i.e., expected heterozygosity and number of segregating sites from posterior predictive simulations were often lower than observed values) and less population structure (numbers of private sites were higher than observed in posterior predictive simulations).

## 4 DISCUSSION

We used population genomic data in an ABC framework to evaluate multiple competing hypotheses to explain the sources and demographic parameters associated with the secondary spread of Round Gobies around Michigan waters of the Great Lakes and into inland Michigan waterways. The use of genomic data was critical because thousands of SNP loci allowed detection of subtle signals of colonization history, despite short timescales (<15 generations) since the initial introduction of the species into the Great Lakes and associated inland waters. The quality of inferences from ABC was assessed using cross‐validation analyses, which quantified the power for ABC model selection and the accuracy and precision of parameter estimates. Cross‐validation results indicated that invasion routes (e.g., upstream or downstream, multiple introductions) in the Great Lakes nearshore habitats and into inland waters could be reliably inferred. However, model selection was unable to always distinguish source populations. Strong support for specific source populations was indicated for the Flint River, which allowed robust estimation of demographic parameters associated with founding events (*N*_e_, bottleneck severity). In other inland systems, model selection supported general model features (e.g., multiple introductions into the Au Sable River system), though power was insufficient to identify a single source population. Broadly, the model identifiability dynamics in these analyses were consistent with previous research that noted the difficulty in distinguishing structurally similar models in ABC analyses (Cabrera & Palsbøll, 2017). Overall, our results show that ABC provides a flexible framework for reconstructing recent colonization histories for nascent invaders that, when coupled with population genomic datasets, can provide robust inference of certain aspects of invasion history.

### 4.1 Colonization histories of inland waterways

Findings from the Lower Peninsula analysis support earlier reports based on an unsupervised clustering (STRUCTURE) analysis (Snyder & Stepien, 2016) that indicated LKM Round Goby were genetically differentiated from collections in Lake STC, and LKE. Bottleneck severity parameter estimates in the present study suggest a moderate reduction in effective population size during the initial spread of Round Goby into Lakes Michigan and Huron (estimated at 15% of contemporary *N*_e_ in this study). In addition, effective size estimates for contemporary Great Lake populations were large but spatially variable. Broadly, the ABC analysis reported here supports the hypothesis that colonization of Michigan's nearshore waters of the Great Lakes by Round Goby was bidirectional from Lake STC and LKM origins. Results from this ABC analysis were used to inform the structure of candidate models for the Cheboygan River, which included potential sources in both Lakes Michigan and Huron.

In the Flint River, the two well‐supported models indicated the system was founded by Round Gobies from SAB. Both models also indicated that initial colonization of the Flint River involved a severe population bottleneck. This could explain both the low diversity and high population divergence as previously noted by other authors (Bronnenhuber et al., 2011; Johansson et al., 2018) and observed as part of this study. The supported models differed in inferences concerning system colonization events. The local model (Local_SAB) infers that humans (potentially anglers) moved Round Gobies upstream above dams in a stepwise manner, while the SAB_HWR model indicates a greater geographic overland movement and single introduction into Holloway Reservoir from SAB followed by downstream dispersal of Round Gobies to uninhabited river segments below the Holloway Reservoir dam. While both models infer “local” (e.g., SAB) sources, either model could indicate invasions were mediated by local angler‐assisted movements or by the commercial bait industry, as has been documented in other systems (Drake & Mandrak, 2010). Similarities in competing model structure likely affect our inability to distinguish them (Cabrera & Palsbøll, 2017) using model selection or cross‐validation. Additional work could further evaluate these hypotheses based on their predictions of the relative effective population sizes among segments (Local_SAB—Saginaw River population smaller than Holloway Reservoir, SAB_HWR—Saginaw River population larger than Holloway Reservoir).

In the Au Sable River, model support indicated that multiple introductions were involved. Despite some uncertainty in model selection (equivocal support for alternative source populations; Figure 3), we evaluated the consistency of parameter estimates obtained under alternative models receiving support (Robinson, Bunnefeld et al., 2014). The bottleneck severity parameter posterior distributions across the supported models indicate weaker founding bottlenecks than estimated for the Flint River. There are several possible explanations for why ABC inference of source population was not strongly supported. First, as noted above, the similarity among candidate models may reduce model identifiability (Cabrera & Palsbøll, 2017). Additionally, the supported models indicate the possibility of considerable migration into the system from multiple sources and subsequent admixture. Furthermore, several features of the parameterized models, including multiple introduction events into the system and a lack of a strong bottleneck associated with colonization, could contribute to the retention of diversity in the Au Sable River populations relative to populations sampled from inland segments of the Flint River system. Given that *N*_e_ priors performed reasonably well in other ABC analysis, it seems likely that the similarity in alternative models, short timescale of the invasion (i.e., limited genetic differentiation of potential source populations), and influx of diversity into the Au Sable from multiple founding events interact to limit our ability to reliably identify source populations.

In the Cheboygan River, we found support for Round Gobies invading the system through the lock (LocalEast) or via long‐distance movement from Saginaw Bay (SAB_MLL), potentially associated with bait release from transient anglers. Differences in contemporary *N*_e_ for Mullett Lake between the two supported models could be used to test their relative support in subsequent analyses. Such analyses were not completed as part of this study because we did not collect additional, independent genotypic information to estimate contemporary *N*_e_. Importantly, both supported Cheboygan River models involve colonization of Mullett Lake before Burt Lake, providing corroborating evidence for this specific colonization pattern.

Results indicate different Round Goby colonization dynamics across three Michigan drainages. Secondary inland spread dynamics into the Flint River system clearly differed from invasions into the Au Sable and Cheboygan Rivers. The Flint River invasion appears to have involved a more severe bottleneck associated with colonization (reduction to <3% of contemporary *N*_e_) compared to the Au Sable and Cheboygan Rivers (~7%). Additionally, colonization of the Au Sable River was likely mediated by multiple introductions into the system. Saginaw Bay in Lake Huron is a plausible source in all three inland systems, which is particularly interesting because it is the source of the majority of the commercial bait in Michigan (T. Goniea, Michigan DNR, personal communication, 2018). Therefore, it is possible that inland invasions were mediated by movements associated with collection and transport of commercial bait. However, ABC model selection analyses were more successful in distinguishing models with alternative invasion routes within each system than alternative source populations. Following initial colonization, subsequent spread of Round Goby was likely achieved by local movements (via natural dispersal and/or local bait movement) among connected portions of inland systems (e.g., from Mullett lake to Burt lake, downstream from Holloway Reservoir).

Posterior predictive simulations were conducted as post hoc assessments of the goodness of fit of the parameterized models for each system. These simulations yielded distributions of statistics that overlapped with our observed values for a majority of the summary statistics used for model selection and parameter estimation; however, some population‐specific statistics (particularly those related to levels of diversity) consistently fell outside of posterior predictive distributions. These discrepancies may be the result of the large number of pairwise statistics used in our analyses. In other words, the population‐specific statistics showing discrepancies with posterior predictive distributions are vastly outnumbered by pairwise statistics, which may have inflated the role of pairwise statistics in parameter estimation. We also note that the use of hyperparameters in our model (for the effective sizes of Great Lakes populations) adds a layer of stochasticity to posterior predictive simulations, which may have impacted goodness‐of‐fit assessments.

## 5 CONCLUSIONS

Testing competing hypotheses concerning aquatic invasive species colonization history using population genetic summary statistics in an ABC framework has become increasingly common. However, many studies have evaluated colonization histories that span broad spatial and temporal scales, which help to amplify signal in the data and facilitate identification of source populations and estimation of associated demographic parameters. The application of SNP data from reduced representation genomic sequencing libraries in an ABC framework is an area of active research (Robinson, Bunnefeld et al., 2014; Shafer et al., 2015). Here, from a genomics perspective, we used SNP data generated from reduced representation libraries and ABC to reconstruct invasion history on a very short time frame (<15 generations). Our results provide crucial insight into the sequence of inland colonization events and estimates of effective population sizes and bottleneck severity in individual systems (along with uncertainty). Importantly, using the ABC framework, we were able to evaluate confidence in inferences being made through cross‐validations and posterior predictive simulations. Despite limited signal and a recent time frame, ABC analyses provide useful inference of routes of inland invasion and, to a lesser extent, possible source populations for Round Goby across three Michigan drainages.

### 5.1 Management implications

Modeling outcomes indicate a pattern of stratified dispersal during inland invasions, defined as an initial colonization event in each system (likely facilitated by human‐assisted movements) followed by natural dispersal. Conceptually, the invasion patterns described here are similar to patterns previously suggested for the Round Goby invasion of the Great Lakes (Brown & Stepien, 2009; LaRue et al., 2011; Snyder & Stepien, 2016) and their tributaries (Bronnenhuber et al., 2011). The stratified dispersal hypothesis has repeatedly been suggested as a mode of movement for other invasive species, including crayfish (Puth & Allen, 2005), multiple native and invasive plant species (Myers, Vellend, Gardescu, & Marks, 2004), mussels (Heiler et al., 2013), insects (Muirhead et al., 2006; Suarez, Holway, & Case, 2001), and fish (Johnson et al., 2008).

The ABC analyses conducted here enabled elimination of specific sources or routes for each invasion (despite little structure among potential sources tested, Figure 2) and provided reliable estimates of founding and contemporary *N*_e_ of the populations evaluated. Based on the supported models, inferences of how Round Gobies entered and dispersed through the inland system(s) can be made. Each of these components enables the identification of targeted areas to mitigate against further spread of the species. For instance, SAB source models were supported, at least to some extent, in each analysis, whereas the Alpena, LTB, and Grand Traverse Bay source populations received little support. Findings inform targeted management actions by focusing community outreach in the SAB area more so than in Alpena, LTB or Grand Traverse Bay. Alternatively, Michigan Department of Natural Resources could implement additional regulations to reduce the risk of Round Goby transfer via personal or commercial bait transfers or increase local outreach efforts to educate the public on the deleterious effects associated with invasive species.

From a management perspective, a useful aspect of ABC inference is the estimation of demographic parameters of interest. In this study, based on estimates of bottleneck severity and contemporary effective size, the founding effective size across all three systems and supported models was small (<50). Our findings suggest that the number of Round Goby founding a new inland system is small (tens to hundreds). This suggests anglers are moving small numbers of Round Goby to new systems or that a small number of Round Goby are missed by commercial bait harvesters when filtering invasive species from their catches intended for the baitfish retail market. This threat can be mitigated with restrictive bait use regulations and working with the commercial bait industry to develop aquatic invasive species hazard analysis and critical control point plans.

Broadly, the identification of invasion routes and estimates of *N*_e_ aid in furthering our understanding of colonization and spread dynamics that can be useful for rapid response for new invasive species (e.g., fish or crayfish) that would have similar mechanisms for spread (e.g., bait bucket release, natural dispersal, overland dispersal via recreational equipment). For example, understanding Round Goby spread dynamics in a few infested rivers could inform actions for other rivers if they become invaded. Actions could include bait bans, lock closures, targeted outreach, targeted enforcement initiatives, or installation of control or deterrent technologies (e.g., Parker et al., 2015). Collectively, combining genomic‐scale datasets in an ABC framework provides useful information to resource agencies regarding historic, recent, and potential future species invasions.

## CONFLICT OF INTEREST

None declared.

## DATA ARCHIVING STATEMENT

Genotypic data used as part of this study are openly available at the Dryad Digital Repository: https://doi.org/10.5061/dryad.2v7s52d. R code used to calculate summary statistics is openly available at the Github repository: nicksard/2019_Goby_ABC_scripts. As an example, the GitHub repository also includes R code to run the Flint River simulations as well.
