# Supplementary material for: Coalescent models characterize sources and demographic history of recent round goby colonization of Great Lakes and inland waters
Source: Evol Appl. 2019 Mar 23;12(5):1034–49. doi: 10.1111/eva.12779 (PMC6503821; doi:10.1111/eva.12779)
Supplement: Supplementary file 1 [file EVA-12-1034-s001.docx]

**Additional details regarding sequencing, bioinformatics, and model development**

*Sequencing and bioinformatics*

DNA was extracted from tissue samples collected at each location using Qiagen DNeasy kits (Qiagen, Valencia, CA). DNA concentrations were estimated using a PicoGreen assay (Thermo Scientific Molecular BioProducts, Waltham, MA) and samples were diluted to 10 ng/μl for restriction site associated DNA (RAD) sequencing library construction. Six Round Goby libraries were prepared for sequencing using the ‘BEST RAD’ protocol (Ali et al., 2016). In brief, 100 ng of DNA per sample was digested with *SbfI* and biotinylated P1 adapters, containing unique 8-base-pair Hamming barcodes, were ligated to digested fragments. Samples were pooled into six libraries, with each library containing uniquely barcoded samples. Libraries were sonicated using an M220 sonicator (Covaris, MA) for 45 seconds (Peak Power: 50.0, Duty Factor: 20.0, Cycles/Burst: 200) to shear DNAs to an average of 550 base pairs (bp) in length. Fragments of DNA, ligated to P1 adapters, were isolated using streptavidin beads. Isolated RAD-tagged DNAs for each library were cleaned with AMPure beads (Beckman Coulter) and each library was barcoded using a NEBNext Ultra DNA library kit for Illumina (New England Biolabs, MA). Paired-end 150-bp sequencing reads were generated for the six libraries on two lanes of an Illumina HiSeq 2500 (v4 flow cell) at Michigan State University’s Research Technology Support Facility. Given the number of samples per location and the number of locations (Table 1), we did not have enough samples for six complete libraries. We accounted for possible lane effects, by evenly dividing samples per location across the two lanes of sequencing. Finally, three other sampling locations were sequenced as part of our libraries, but were used in another ongoing project and therefore were not evaluated as part of this study.

Sequencing data for all libraries were processed using STACKS v. 1.44 (Catchen et al., 2013; Catchen et al., 2011) using computational resources provided by Michigan State University’s Institute for Cyber-Enabled Research. Initially, duplicate-reads, identical reads that arise during PCR enrichment of adapter-ligated DNAs, were removed from each library using *clone_filter* to avoid inflated coverages that can lead to incorrect genotype calls (Andrews et al., 2014). Libraries were demultiplexed with *process_radtags*. During *process_radtags*, reads containing up to one mismatch between observed and expected barcodes and restriction site sequences were used to identify SNPs in subsequent STACKS functions (Catchen et al. 2013). One mismatch was allowed because we used Hamming barcodes that enable unambiguous identification when a sequence error occurs within the barcode itself. In addition, reads with uncalled bases, as well as low-quality reads (default STACKS value: average phred score of 10 in a 22 bp sliding window across the read) were removed. We used *ustacks* to identify putative loci within each individual, setting the minimum number of stacks to form an allele (-m) to three and the maximum distance between alleles to form a putative locus (-M) to two, which could result from mutation or sequencing error. The deleveraging algorithm was used to resolve over merged stacks. Two individuals from each sampled location were used when forming the catalog in *cstacks* and allowed for a maximum of two nucleotide differences between stacks from different individuals (-n) when identifying loci genotyped in multiple individuals. Each set of loci within a sample was compared to the catalog using *sstacks*. We used *populations* to call SNP genotypes for all sequenced individuals. We only used the first bi-allelic SNP observed on the RAD-tag, and did not consider the entire tag as a “mini-haplotype”. No minor allele frequency cutoff was used because we expect any new mutation that has occurred since the initial invasion of Round Gobies is likely rare and represented in only a single or few sites. These data may be important for identifying source populations (via pairwise-private sites, see explanation in Methods, and average pairwise private site frequency calculations) performed as part of our analysis. Including a minimum minor allele frequency filter during genotype calling would therefore impose a form of ascertainment bias, which would need to be modeled in our coalescent simulations. Instead, we include the first variable site observed per 150 bp RAD locus in downstream analyses. Loci were included in the final VCF file if they were successfully genotyped in 80% of the individuals within a population at a depth of ≥ 9x coverage and were genotyped in 15 of the 18 populations sampled, which resulted in a file containing 7,802 loci. The above parameters were chosen to maximize the number of loci genotyped for each ABC analysis while minimizing the risk of genotyping errors (Mastretta-Yates et al., 2015, Buerkle et al., 2013, Fountain et al., 2016).

*Flint River model development example*

For the Flint River system, one plausible model would involve the movement of Round Gobies from Saginaw Bay into Holloway Reservoir (potentially via bait bucket transfer). Figure A1.1a provides a simplified graphical depiction of this model in which each branching event on the tree represents the colonization of a new population from an existing (source) population. Starting from the bottom of the tree, the St. Clair River and Lake St. Clair were colonized by Round Gobies originally from the native range (Black Sea) prior to 1990 (time 1). By 1993 (time 2), Round Gobies had been detected in both Lake Erie and Lake Michigan, likely as a result of movement in ballast tanks of container ships operating within the Great Lakes. At time 3, migrants from the Lake St. Clair population colonize a population in Saginaw Bay. Thereafter, individuals from the Saginaw Bay population spread to unimpounded segments of the Saginaw River (time 4) and to Holloway Reservoir, possibly by angler-assisted movement (time 5). Following the invasion into Holloway Reservoir, the remainder of the Flint system is colonized in a downstream, stepping-stone fashion, with movements from Holloway Reservoir to Mott Lake (time 6) and from Mott Lake to the impounded segment below Mott Lake (BML; time 7). All models considered for the Flint system share the early portion of this history (times 1 to 4, representing branching events), but differed in the source population that provided colonists to the Flint River system, and in the order of colonization events within the impounded portions of the Flint River (Figure A1). That is, the Flint River system could have been founded by Round Gobies from Lake Michigan, Lake Erie, Lake St. Clair, or Saginaw Bay (Figure A1.1b). In addition, there are multiple hypotheses to explain the nature in which the system was colonized, as the river is segmented by three dams. Based on USGS records (Fuller et al., 2018) of the Round Goby invasion in the system, it is possible that Round Gobies: 1) initially founded Holloway Reservoir and then colonized the system in a downstream stepping-stone process, 2) initially founded Mott Lake and Round Gobies subsequently spread upstream to Holloway Reservoir via angler-assisted movements, and downstream via natural dispersal, or 3) Holloway Reservoir and Mott Lake were colonized by separate introductions from the same source, and the segment of the Flint River below Mott Lake was colonized by subsequent natural dispersal (Figure A1.1c). Tables A1.1- A1.4 provide detailed descriptions of the order of events and source populations involved in each simulated model for each ABC analysis.

*ABC model development*

Beyond a similar overall structure and early invasion history, simulated models shared several other features across systems. Briefly, we allowed continuous migration among potential source populations associated with shipping ports (SHP) (e.g., St. Clair system, Saginaw Bay, Rogers City, etc.) based on several studies that have demonstrated connectivity among ports within the Great Lakes (Johansson et al., 2018; LaRue et al., 2011). Shipping-assisted migration was assumed to have occurred at an equal rate between all pairs of ports. We also allowed migration between populations in the Great Lakes (e.g., Saginaw Bay, LMIG) and between Great Lakes populations and those occupying adjacent unimpounded portions of tributaries (e.g., between Saginaw Bay and the Saginaw River, MBR). Additionally, downstream (and in one model, upstream) migration within the inland systems (MDS / MUS), and migration between colonized inland segments and their respective source populations (i.e., bait bucket migration, BBM) were also incorporated into the models. Parameters for colonization bottleneck severity (proportional reduction in effective size relative to contemporary N_e_) were assumed to be equal for all shipping-mediated colonization events (i.e., early colonization of Lake Michigan and Lake Erie populations, SF - Source Founding severity) and for colonization of inland segments of each drainage system (RF - River Founding severity). For the Cheboygan system models only, the founding size for the upper lake populations (without ports - LTB, GTB, and Cheboygan; NF - Natural Founding severity) was modeled separately from the bottleneck severity in other Great Lakes populations because colonization processes associated with natural dispersal (not associated with ballast water movements) may result in a different degree of bottleneck severity. We excluded LTB and GTB as potential sources for Flint and Au Sable analyses because of the large geographic distances separating these populations from the inland segments and their comparatively late appearance in the USGS database (Fuller et al., 2018). A separate parameter was used to model the initial colonization of the St. Clair system from the native population in the Black Sea (IF - Invasion Founding severity). Prior distributions for all model parameters were chosen by evaluating previous genetic studies (Bronnenhuber et al., 2011; Brown and Stepien 2009; Johansson et al. 2018; LaRue et al., 2011, Snyder and Stepien 2016) and studies detailing Round Goby life history (reviewed by Kornis et al., 2012, and references therein) and were broad such that a wide range of plausible values could be drawn (Tables A1.5 - A1.8).

*References*

Ali, O. A., O'Rourke, S. M., Amish, S. J., Meek, M. H., Luikart, G., Jeffres, C., & Miller, M. R. (2016). RAD Capture (Rapture): Flexible and Efficient Sequence-Based Genotyping. *Genetics, 202*(2), 389-400. doi:10.1534/genetics.115.183665

Andrews, K. R., & Luikart, G. (2014). Recent novel approaches for population genomics data analysis. *Molecular Ecology, 23*, 1661-1667.

Bronnenhuber, J. E., Dufour, B. A., Higgs, D. M., & Heath, D. D. (2011). Dispersal strategies, secondary range expansion and invasion genetics of the nonindigenous round goby, *Neogobius melanostomus*, in Great Lakes tributaries. *Molecular Ecology, 20*(9), 1845-1859. doi:10.1111/j.1365-294X.2011.05030.x

Brown, J. E., & Stepien, C. A. (2009). Invasion genetics of the Eurasian round goby in North America: tracing sources and spread patterns. *Molecular Ecology, 18*(1), 64-79. doi:10.1111/j.1365-294X.2008.04014.x

Buerkle, C. and Gompert, Z., 2013. Population genomics based on low coverage sequencing: how low should we go?. Molecular Ecology, 22(11), pp.3028-3035.

Catchen, J., Hohenlohe, P. A., Bassham, S., Amores, A., & Cresko, W. A. (2013). Stacks: an analysis tool set for population genomics. *Molecular Ecology, 22*(11), 3124-3140. doi:10.1111/mec.12354

Catchen, J. M., Amores, A., Hohenlohe, P., Cresko, W., & Postlethwait, J. H. (2011). Stacks: building and genotyping Loci de novo from short-read sequences. *G3 (Bethesda), 1*(3), 171-182. doi:10.1534/g3.111.000240

Fountain, E.D., Pauli, J.N., Reid, B.N., Palsbøll, P.J. and Peery, M.Z., 2016. Finding the right coverage: the impact of coverage and sequence quality on single nucleotide polymorphism genotyping error rates. Molecular ecology resources, 16(4), pp.966-978.

Fuller, P., Benson, A., Maynard, E., Neilson, M. E., Larson, J., & Fusaro, A. (2018). *Neogobius melanostomus* (Pallas, 1814): U.S. Geological Survey, Nonindigenous Aquatic Species Database. Retrieved from <https://nas.er.usgs.gov/queries/factsheet.aspx?SpeciesID=713>

Johansson, M. L., Dufour, B. A., Wellband, K. W., Corkum, L. D., MacIsaac, H. J., & Heath, D. D. (2018). Human-mediated and natural dispersal of an invasive fish in the eastern Great Lakes. *Heredity, 120*(6), 533-546.

Kornis, M. S., Mercado-Silva, N., & Vander Zanden, M. J. (2012). Twenty years of invasion: a review of round goby *Neogobius melanostomus* biology, spread and ecological implications. *Journal of Fish Biology, 80*(2), 235-285. doi:10.1111/j.1095-8649.2011.03157.x

LaRue, E. A., Ruetz, C. R., Stacey, M. B., & Thum, R. A. (2011). Population genetic structure of the round goby in Lake Michigan: implications for dispersal of invasive species. *Hydrobiologia, 663*(1), 71-82.

Mastretta‐Yanes, A., Arrigo, N., Alvarez, N., Jorgensen, T.H., Piñero, D. and Emerson, B.C., 2015. Restriction site‐associated DNA sequencing, genotyping error estimation and de novo assembly optimization for population genetic inference. *Molecular Ecology Resources*, *15*(1), pp.28-41.

Snyder, M. R., & Stepien, C. A. (2016). Genetic patterns across an invasion's history: a test of change versus stasis for the Eurasian round goby in North America. *Molecular Ecology*. doi:10.1111/mec.13997

Table A1.1. Summary of the four models used to test how the Round Goby colonized the Great Lakes around the Lower Peninsula of Michigan. The ‘Model’ column display abbreviated model names that represent the last location to trace its linage back through Lake Huron to Lake St. Clair. Each Time event represent branching events on the trees – i.e. new founding events. For instance in Time 1, Lake St. Clair was founded by individuals from the native range (NAT > STC). Times with an ‘&’ symbol represent polytomies, with multiple founding events occurring at the same time. All four models share the same branching events in Times 1 – 3. Abbreviations for locations are as follows: NAT – Native source, LKM – Lake Michigan, STC – Lake St. Clair, LKE – Lake Erie, SAB – Saginaw Bay, ALP – Alpena, MGL – Muskegon Lake, RGC – Roger City, CBR – Cheboygan River, LTB – Little Traverse Bay, GTB – Grand Traverse Bay.

| **Model** | **Time 1** | **Time 2** | **Time 3** | **Time 4** | **Time 5** | **Time 6** | **Time 7** | **Time 8** |
| --- | --- | --- | --- | --- | --- | --- | --- | --- |
| **CBR** | NAT > STC | STC > LKM & STC > LKE | STC > SAB & STC > ALP | LKM > MGL  LKM > MGL  LKM > MGL | ALP > RGC | RGC > CBR | MGL > GTB | GTB > LTB |
| **LTB** |  |  |  |  | ALP > RGC | RGC > CBR | CBR > LTB | MGL > GTB |
| **GTB** |  |  |  |  | ALP > RGC | RGC > CBR | CBR > LTB | LTB > GTB |
| **MGL** |  |  |  | ALP > RGC | RGC > CBR | CBR > LTB | LTB > GTB | GTB > MGL |

Table A1.2. Summary of the thirteen models used to test how the Round Goby colonized the Flint River basin in Michigan. The ‘Model’ column display abbreviated model names that represent the source of the introduction (name to the left of the underscore), and the first location(s) that were founded with the system (to the right of the underscore). MLL+HWR represents introductions into both Mott Lake and Holloway Reservoir at the same time. ‘Local’ models represent upstream stepping-stone models via bait-bucket movements. LocalSAB represents Round Gobies collected as bait in Saginaw River that were brought into the system and then moved upstream. Each Time event represent branching events on the trees – i.e. new founding events. For instance in Time 1, Lake St. Clair was founded by individuals from the native range (NAT > STC). Times with an ‘&’ symbol represent polytomies, with multiple founding events occurring at the same time. All thirteen models share the same branching events in Times 1 – 4. Abbreviations for locations are as follows: NAT – Native source, LKM – Lake Michigan, STC – Lake St. Clair, LKE – Lake Erie, SAB – Saginaw Bay, SAR – Saginaw River, BML – Below Mott Lake, MTL – Mott Lake, HWR – Holloway Reservoir.

| **Model** | **Time 1** | **Time 2** | **Time 3** | **Time 4** | **Time 5** | **Time 6** | **Time 7** |
| --- | --- | --- | --- | --- | --- | --- | --- |
| **LocalSAB** | NAT > STC | STC > LKE & STC > LKM | STC > SAB | SAB > SAR | SAR > BML | BML > MTL | MTL > HWR |
| **SAB_HWR** |  |  |  |  | SAB > HWR | HWR > MTL | MTL > BML |
| **SAB_MTL+HWR** |  |  |  |  | SAB > MTL | SAB > HWR | MTL > BML |
| **SAB_MTL** |  |  |  |  | SAB > MTL | MTL > BML | MTL > HWR |
| **LKE_HWR** |  |  |  |  | LKE > HWR | HWR > MTL | MTL > BML |
| **LKE_MTL+HWR** |  |  |  |  | LKE > MTL | SAB > HWR | MTL > BML |
| **LKE_MTL** |  |  |  |  | LKE > MTL | MTL > BML | MTL > HWR |
| **LKM_HWR** |  |  |  |  | LKM > HWR | HWR > MTL | MTL > BML |
| **LKM_MTL+HWR** |  |  |  |  | LKM > MTL | SAB > HWR | MTL > BML |
| **LKM_MTL** |  |  |  |  | LKM > MTL | MTL > BML | MTL > HWR |
| **STC_HWR** |  |  |  |  | STC > HWR | HWR > MTL | MTL > BML |
| **STC_MTL+HWR** |  |  |  |  | STC > MTL | SAB > HWR | MTL > BML |
| **STC_MTL** |  |  |  |  | STC > MTL | MTL > BML | MTL > HWR |

Table A1.3. Summary of the seventeen models used to test how the Round Goby colonized the Au Sable River basin in Michigan. The ‘Model’ column display abbreviated model names that represent the source of the introduction (name to the left of the underscore), and the first location(s) that were founded with the system (to the right of the underscore). CDP+FCP represents introductions into both Cook Dam Pond and Five Channels Pond at the same time. ‘Local’ models represent upstream stepping-stone models via bait-bucket movements. LocalSAB represents Round Gobies collected as bait in Saginaw Bay that were brought into the system and then moved upstream. Each Time event represent branching events on the trees – i.e. new founding events. For instance in Time 1, Lake St. Clair was founded by individuals from the native range (NAT > STC). Times with an ‘&’ symbol represent polytomies, with multiple founding events occurring at the same time. All seventeen models share the same branching events in Times 1 – 3. Abbreviations for locations are as follows: NAT – Native source, LKM – Lake Michigan, STC – Lake St. Clair, LKE – Lake Erie, SAB – Saginaw Bay, ALP – Alpena, ASL – Au Sable River mouth, FDP – Foote Dam Pond, CDP – Cook Dam Pond, FCP – Five Channels Pond.

| **Model** | **Time 1** | **Time 2** | **Time 3** | **Time 4** | **Time 5** | **Time 6** | **Time 7** |
| --- | --- | --- | --- | --- | --- | --- | --- |
| **LocalALP** | NAT > STC | STC > LKE & STC > LKM | STC > SAB & STC > ALP | ALP > ASL | ASL > FDP | FDP > CDP | CDP > FCP |
| **LocalSAB** |  |  |  | SAB > ASL | ASL > FDP | FDP > CDP | CDP > FCP |
| **SAB_FCP** |  |  |  | SAB > ASL | SAB > FCP | FCP > CDP | CDP > FDP |
| **SAB_CDP+FCP** |  |  |  | SAB > ASL | SAB > CDP | SAB > FCP | CDP > FDP |
| **SAB_CDP** |  |  |  | SAB > ASL | SAB > CDP | CDP > FDP | CDP > FCP |
| **LKE_FCP** |  |  |  | SAB > ASL | LKE > FCP | FCP > CDP | CDP > FDP |
| **LKE_CDP+FCP** |  |  |  | SAB > ASL | LKE > CDP | LKE > FCP | CDP > FDP |
| **LKE_CDP** |  |  |  | SAB > ASL | LKE > CDP | CDP > FDP | CDP > FCP |
| **LKM_FCP** |  |  |  | SAB > ASL | LKM > FCP | FCP > CDP | CDP > FDP |
| **LKM_CDP+FCP** |  |  |  | SAB > ASL | LKM > CDP | LKM > FCP | CDP > FDP |
| **LKM_CDP** |  |  |  | SAB > ASL | LKM > CDP | CDP > FDP | CDP > FCP |
| **STC_FCP** |  |  |  | SAB > ASL | STC > FCP | FCP > CDP | CDP > FDP |
| **STC_CDP+FCP** |  |  |  | SAB > ASL | STC > CDP | STC > FCP | CDP > FDP |
| **STC_CDP** |  |  |  | SAB > ASL | STC > CDP | CDP > FDP | CDP > FCP |
| **ALP_FCP** |  |  |  | SAB > ASL | ALP > FCP | FCP > CDP | CDP > FDP |
| **ALP_CDP+FCP** |  |  |  | SAB > ASL | ALP > CDP | ALP > FCP | CDP > FDP |
| **ALP_CDP** |  |  |  | SAB > ASL | ALP > CDP | CDP > FDP | CDP > FCP |

Table A1.4. Summary of the twenty-one models used to test how the Round Goby colonized the Cheboygan River basin in Michigan. The ‘Model’ column display abbreviated model names that represent the source of the introduction (name to the left of the underscore), and the first location(s) that were founded with the system (to the right of the underscore). BTL+MLL represents introductions into both Burt Lake and Mullett Lake at the same time. ‘Local’ models represent upstream stepping-stone models via bait-bucket movements. LocalEAST represents Round Gobies collected as bait in the Cheboygan River that were brought into the system from east to west. Each Time event represent branching events on the trees – i.e. new founding events. For instance in Time 1, Lake St. Clair was founded by individuals from the native range (NAT > STC). Times with an ‘&’ symbol represent polytomies, with multiple founding events occurring at the same time. All seventeen models share the same branching events in Times 1 – 8. Abbreviations for locations are as follows: NAT – Native source, LKM – Lake Michigan, STC – Lake St. Clair, LKE – Lake Erie, SAB – Saginaw Bay, ALP – Alpena, MGL – Muskegon Lake, RGC – Roger City, CBR – Cheboygan River, LTB – Little Traverse Bay, GTB – Grand Traverse Bay, MLL – Mullett Lake, and BTL – Burt Lake.

| **Model** | **Time 1** | **Time 2** | **Time 3** | **Time 4** | **Time 5** | **Time 6** | **Time 7** | **Time 8** | **Time 9** | **Time 10** |
| --- | --- | --- | --- | --- | --- | --- | --- | --- | --- | --- |
| **LocalEAST** | NAT > STC | STC > LKM & STC > LKE | STC > SAB & STC > ALP | LKM > MGL | ALP > RGC | RGC > CBR | MGL > GTB | GTB > LTB | CBR > MLL | MLL > BTL |
| **LocalWEST** |  |  |  |  |  |  |  |  | LTB > BTL | BTL > MLL |
| **LocalBOTH** |  |  |  |  |  |  |  |  | CBR > MLL | LTB > BTL |
| **LKE_BTL** |  |  |  |  |  |  |  |  | LKE > BTL | BTL > MLL |
| **LKE_BTL+MLL** |  |  |  |  |  |  |  |  | LKE > MLL | LKE > BTL |
| **LKE_MLL** |  |  |  |  |  |  |  |  | LKE > MLL | MLL > BTL |
| **LKM_BTL** |  |  |  |  |  |  |  |  | LKM > BTL | BTL > MLL |
| **LKM_BTL+MLL** |  |  |  |  |  |  |  |  | LKM > MLL | LKM > BTL |
| **LKM_MLL** |  |  |  |  |  |  |  |  | LKM > MLL | MLL > BTL |
| **STC_BTL** |  |  |  |  |  |  |  |  | STC > BTL | BTL > MLL |
| **STC_BTL+MLL** |  |  |  |  |  |  |  |  | STC > MLL | STC > BTL |
| **STC_MLL** |  |  |  |  |  |  |  |  | STC > MLL | MLL > BTL |
| **SAB_BTL** |  |  |  |  |  |  |  |  | SAB > BTL | BTL > MLL |
| **SAB_BTL+MLL** |  |  |  |  |  |  |  |  | SAB > MLL | SAB > BTL |
| **SAB_MLL** |  |  |  |  |  |  |  |  | SAB > MLL | MLL > BTL |
| **ALP_BTL** |  |  |  |  |  |  |  |  | ALP > BTL | BTL > MLL |
| **ALP_BTL+MLL** |  |  |  |  |  |  |  |  | ALP > MLL | ALP > BTL |
| **ALP_MLL** |  |  |  |  |  |  |  |  | ALP > MLL | MLL > BTL |
| **GTB_BTL** |  |  |  |  |  |  |  |  | GTB > BTL | BTL > MLL |
| **GTB_BTL+MLL** |  |  |  |  |  |  |  |  | GTB > MLL | GTB > BTL |
| **GTB_MLL** |  |  |  |  |  |  |  |  | GTB > MLL | MLL > BTL |

Table A1.5. Summary of the prior distributions for parameters used in the Lower Peninsula analysis.

| **Type** | **Parameter** | **Distribution** | **Min** | **Max** |
| --- | --- | --- | --- | --- |
| Ne | LAKEMEAN | loguniform | 500 | 10000 |
|  | LAKECV | uniform | 0.05 | 0.5 |
| Migration | SHP | uniform | 0.0001 | 0.001 |
|  | LMIG | uniform | 0.0001 | 0.001 |
| Bottleneck | SF | uniform | 0.01 | 0.2 |
|  | IF | uniform | 0.1 | 0.4 |
|  | NF | uniform | 0.01 | 0.1 |

Table A1.6. Summary of the prior distributions for parameters used in the Flint River analysis.

| **Type** | **Parameter** | **Distribution** | **Min** | **Max** |
| --- | --- | --- | --- | --- |
| Ne | LAKEMEAN | loguniform | 500 | 10000 |
|  | LAKECV | uniform | 0.05 | 0.5 |
|  | SAR | loguniform | 50 | 1000 |
|  | BML | loguniform | 50 | 1000 |
|  | MTL | loguniform | 50 | 1000 |
|  | HWR | loguniform | 50 | 1000 |
| Migration | SHP | uniform | 0.0001 | 0.001 |
|  | MBR | uniform | 0.0001 | 0.001 |
|  | MDS | uniform | 0.00001 | 0.001 |
|  | MUS | loguniform | 0.000005 | 0.0005 |
|  | BBM | loguniform | 0.000005 | 0.0005 |
| Bottleneck | RF | uniform | 0.001 | 0.1 |
|  | SF | uniform | 0.01 | 0.2 |
|  | IF | uniform | 0.1 | 0.4 |

Table A1.7. Summary of the prior distributions for parameters used in the Au Sable River analysis.

| **Type** | **Parameter** | **Distribution** | **Min** | **Max** |
| --- | --- | --- | --- | --- |
| Ne | LAKEMEAN | loguniform | 500 | 10000 |
|  | LAKECV | uniform | 0.05 | 0.5 |
|  | LAS | loguniform | 50 | 1000 |
|  | FDP | loguniform | 50 | 1000 |
|  | CDP | loguniform | 50 | 1000 |
|  | FCP | loguniform | 50 | 1000 |
| Migration | SHP | uniform | 0.0001 | 0.001 |
|  | MBR | uniform | 0.0001 | 0.001 |
|  | MDS | uniform | 0.00001 | 0.001 |
|  | MUS | loguniform | 0.000005 | 0.0005 |
|  | BBM | loguniform | 0.000005 | 0.0005 |
| Bottleneck | RF | uniform | 0.001 | 0.1 |
|  | SF | uniform | 0.01 | 0.2 |
|  | IF | uniform | 0.1 | 0.4 |

Table A1.8. Summary of the prior distributions for parameters used in the Cheboygan River analysis.

| **Type** | **Parameter** | **Distribution** | **Min** | **Max** |
| --- | --- | --- | --- | --- |
| Ne | LAKEMEAN | loguniform | 500 | 10000 |
|  | LAKECV | uniform | 0.05 | 0.5 |
|  | MLL | loguniform | 50 | 1000 |
|  | BTL | loguniform | 50 | 1000 |
| Migration | SHP | uniform | 0.0001 | 0.001 |
|  | LMIG | uniform | 0.0001 | 0.001 |
|  | MDS | uniform | 0.00001 | 0.001 |
|  | MBM | uniform | 0.0001 | 0.001 |
|  | BBM | loguniform | 0.000005 | 0.0005 |
| Bottleneck | RF | uniform | 0.001 | 0.1 |
|  | SF | uniform | 0.01 | 0.2 |
|  | IF | uniform | 0.1 | 0.4 |
|  | NF | uniform | 0.01 | 0.1 |

*
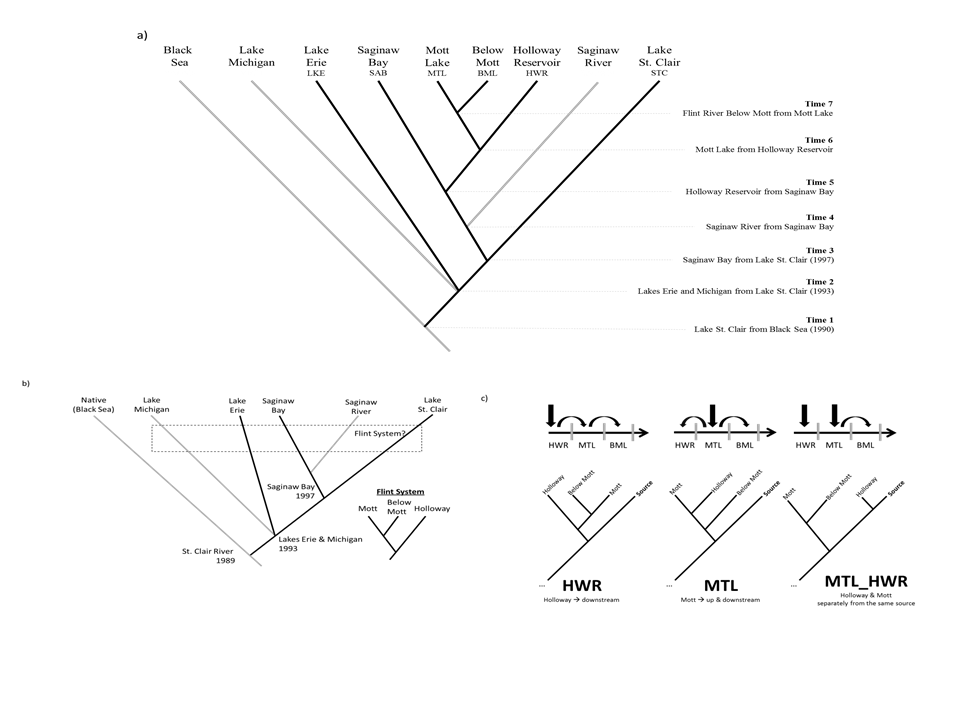
*

Figure A1.1. Graphical representation of hypothesis development in the Flint River system. Figure A1.1a depicts one model to explain the invasion of the Flint River system that was tested. Light gray lines indicate unsampled populations (i.e., populations that were simulated in models, but that lacked observed genotypic information). Figure A1.1b depicts a tree representing the colonization of the Great Lakes. The dotted rectangle represents the possible placements of the Flint River system. Finally, Figure A1.1c depicts three possible colonization mechanisms within the Flint River itself: Holloway Reservoir (HWR), Mott Lake (MTL), and the impounded segment of the Flint River below Mott Lake (BML). Horizontal lines represent the Flint River, and perpendicular grey lines represent impoundments that prevent natural dispersal. Vertical, downward facing arrows represent the initial founding event(s).
