# Supplementary material for: Coalescent models characterize sources and demographic history of recent round goby colonization of Great Lakes and inland waters
Source: Evol Appl. 2019 Mar 23;12(5):1034–49. doi: 10.1111/eva.12779 (PMC6503821; doi:10.1111/eva.12779)
Supplement: Supplementary file 2 [file EVA-12-1034-s002.docx]

**Appendix 2 – Parameter estimation and power assessment.** This appendix provides additional tables and figures associated with the results of each approximate Bayesian Computation analysis.

Table A2.1. For the Lower Peninsula random forest analysis, the proportion of assignments to the correct model are represented on the diagonal of the table; whereas, the out-of-bag error rates represent the proportion of datasets misassigned to alternative models are represented in the remainder of the table. The first column and row (in bold) display abbreviated model names that represent the last location to trace its linage back through Lake Huron to Lake St. Clair. Abbreviations for locations are as follows: MGL – Muskegon Lake, CBR – Cheboygan River, LTB – Little Traverse Bay, GTB – Grand Traverse Bay. See Table A1.1 (Appendix 1) for description of models.

|  | **CBR** | **GTB** | **LTB** | **MGL** |
| --- | --- | --- | --- | --- |
| **CBR** | 0.993 | 0.002 | 0.001 | 0.004 |
| **GTB** | 0.002 | 0.995 | 0.002 | 0.002 |
| **LTB** | 0.001 | 0.002 | 0.996 | 0.002 |
| **MGL** | 0.003 | 0.002 | 0.001 | 0.994 |

Table A2.2. For the Flint River random forest analysis, the proportion of assignments to the correct model are represented on the diagonal of the table; whereas, the out-of-bag error rates represent the proportion of datasets misassigned to alternative models are represented in the remainder of the table. The first column and row (in bold) display abbreviated model names that represent the source of the introduction (name to the left of the underscore), and the first location(s) that were founded within the system (to the right of the underscore). MLL+HWR represents introductions into both Mott Lake and Holloway Reservoir at the same time. ‘Local’ models represent upstream stepping-stone models via bait-bucket movements. LocalSAB represents Round Gobies collected as bait in Saginaw River that were brought into the system and then moved upstream. Abbreviations for locations are as follows: LKM – Lake Michigan, STC – Lake St. Clair, LKE – Lake Erie, SAB – Saginaw Bay, BML – Below Mott Lake, MTL – Mott Lake, HWR – Holloway Reservoir. See Table A1.2 (Appendix 1) for description of models.

|  | **LKE_HWR** | **LKE_MTL+HWR** | **LKE_MTL** | **LocalSAB** | **LKM_HWR** | **LKM_MTL+HWR** | **LKM_MTL** | **SAB_HWR** | **SAB_MTL+HWR** | **SAB_MTL** | **STC_HWR** | **STC_MTL+HWR** | **STC_MTL** |
| --- | --- | --- | --- | --- | --- | --- | --- | --- | --- | --- | --- | --- | --- |
| **LKE_HWR** | 0.769 | 0.001 | 0.017 | 0.002 | 0.086 | 0.001 | 0.002 | 0.066 | 0.000 | 0.001 | 0.055 | 0.000 | 0.001 |
| **LKE_MTL+HWR** | 0.006 | 0.825 | 0.005 | 0.000 | 0.000 | 0.058 | 0.000 | 0.000 | 0.053 | 0.000 | 0.000 | 0.052 | 0.000 |
| **LKE_MTL** | 0.010 | 0.001 | 0.768 | 0.023 | 0.001 | 0.000 | 0.079 | 0.001 | 0.000 | 0.062 | 0.000 | 0.000 | 0.054 |
| **LocalSAB** | 0.000 | 0.000 | 0.001 | 0.981 | 0.000 | 0.000 | 0.004 | 0.000 | 0.000 | 0.013 | 0.000 | 0.000 | 0.001 |
| **LKM_HWR** | 0.167 | 0.000 | 0.002 | 0.003 | 0.438 | 0.001 | 0.011 | 0.159 | 0.000 | 0.002 | 0.214 | 0.000 | 0.002 |
| **LKM_MTL+HWR** | 0.001 | 0.138 | 0.001 | 0.000 | 0.007 | 0.482 | 0.005 | 0.001 | 0.139 | 0.000 | 0.012 | 0.208 | 0.007 |
| **LKM_MTL** | 0.002 | 0.000 | 0.164 | 0.033 | 0.006 | 0.001 | 0.422 | 0.001 | 0.000 | 0.151 | 0.002 | 0.000 | 0.218 |
| **SAB_HWR** | 0.083 | 0.000 | 0.001 | 0.004 | 0.093 | 0.000 | 0.002 | 0.717 | 0.001 | 0.015 | 0.082 | 0.000 | 0.001 |
| **SAB_MTL+HWR** | 0.000 | 0.062 | 0.000 | 0.000 | 0.000 | 0.064 | 0.000 | 0.007 | 0.776 | 0.006 | 0.000 | 0.083 | 0.000 |
| **SAB_MTL** | 0.001 | 0.000 | 0.081 | 0.042 | 0.001 | 0.000 | 0.085 | 0.009 | 0.001 | 0.698 | 0.001 | 0.000 | 0.082 |
| **STC_HWR** | 0.139 | 0.000 | 0.002 | 0.002 | 0.280 | 0.004 | 0.011 | 0.174 | 0.000 | 0.003 | 0.378 | 0.001 | 0.006 |
| **STC_MTL+HWR** | 0.000 | 0.109 | 0.000 | 0.000 | 0.000 | 0.159 | 0.000 | 0.000 | 0.143 | 0.000 | 0.001 | 0.585 | 0.001 |
| **STC_MTL** | 0.001 | 0.000 | 0.135 | 0.031 | 0.005 | 0.001 | 0.268 | 0.002 | 0.000 | 0.166 | 0.004 | 0.001 | 0.385 |

Table A2.3. For the Au Sable River random forest analysis, the proportion of assignments to the correct model are represented on the diagonal of the table; whereas, the out-of-bag error rates represent the proportion of datasets misassigned to alternative models are represented in the remainder of the table. The first column and row (in bold) display abbreviated model names that represent the source of the introduction (name to the left of the underscore), and the first location(s) that were founded within the system (to the right of the underscore). CDP+FCP represents introductions into both Cook Dam Pond and Five Channels Pond at the same time. ‘Local’ models represent upstream stepping-stone models via bait-bucket movements. LocalSAB represents Round Gobies collected as bait in Saginaw Bay that were brought into the system and then moved upstream. Each Time event represent branching events on the trees – i.e. new founding events. Abbreviations for locations are as follows: LKM – Lake Michigan, STC – Lake St. Clair, LKE – Lake Erie, SAB – Saginaw Bay, ALP – Alpena, FDP – Foote Dam Pond, CDP – Cook Dam Pond, FCP – Five Channels Pond. See Table A1.3 (Appendix 1) for description of models.

|  | **ALP_FCP** | **ALP_CDP+FCP** | **ALP_CDP** | **LKE_FCP** | **LKE_CDP+FCP** | **LKE_CDP** | **LocalALP** | **LocalSAB** | **LKM_FCP** | **LKM_CDP+FCP** | **LKM_CDP** | **SAB_FCP** | **SAB_CDP+FCP** | **SAB_CDP** | **STC_FCP** | **STC_CDP+FCP** | **STC_CDP** |
| --- | --- | --- | --- | --- | --- | --- | --- | --- | --- | --- | --- | --- | --- | --- | --- | --- | --- |
| **ALP_FCP** | 0.747 | 0.001 | 0.016 | 0.054 | 0.000 | 0.001 | 0.003 | 0.001 | 0.063 | 0.000 | 0.001 | 0.057 | 0.000 | 0.001 | 0.053 | 0.000 | 0.001 |
| **ALP_CDP+FCP** | 0.006 | 0.808 | 0.005 | 0.000 | 0.041 | 0.000 | 0.000 | 0.000 | 0.000 | 0.042 | 0.000 | 0.000 | 0.047 | 0.000 | 0.000 | 0.050 | 0.000 |
| **ALP_CDP** | 0.008 | 0.001 | 0.712 | 0.000 | 0.000 | 0.051 | 0.054 | 0.008 | 0.001 | 0.000 | 0.058 | 0.000 | 0.000 | 0.055 | 0.000 | 0.000 | 0.052 |
| **LKE_FCP** | 0.051 | 0.000 | 0.001 | 0.774 | 0.001 | 0.018 | 0.001 | 0.001 | 0.064 | 0.000 | 0.002 | 0.045 | 0.000 | 0.001 | 0.040 | 0.000 | 0.001 |
| **LKE_CDP+FCP** | 0.000 | 0.038 | 0.000 | 0.006 | 0.835 | 0.005 | 0.000 | 0.000 | 0.000 | 0.043 | 0.000 | 0.000 | 0.036 | 0.000 | 0.000 | 0.037 | 0.000 |
| **LKE_CDP** | 0.000 | 0.000 | 0.047 | 0.008 | 0.001 | 0.765 | 0.016 | 0.017 | 0.001 | 0.000 | 0.061 | 0.000 | 0.000 | 0.044 | 0.000 | 0.000 | 0.039 |
| **LocalALP** | 0.000 | 0.000 | 0.018 | 0.000 | 0.000 | 0.001 | 0.802 | 0.172 | 0.000 | 0.000 | 0.004 | 0.000 | 0.000 | 0.001 | 0.000 | 0.000 | 0.001 |
| **LocalSAB** | 0.000 | 0.000 | 0.002 | 0.000 | 0.000 | 0.002 | 0.194 | 0.780 | 0.000 | 0.000 | 0.004 | 0.000 | 0.000 | 0.017 | 0.000 | 0.000 | 0.002 |
| **LKM_FCP** | 0.119 | 0.000 | 0.002 | 0.138 | 0.000 | 0.002 | 0.001 | 0.001 | 0.433 | 0.001 | 0.011 | 0.116 | 0.000 | 0.002 | 0.171 | 0.000 | 0.002 |
| **LKM_CDP+FCP** | 0.000 | 0.098 | 0.000 | 0.001 | 0.117 | 0.000 | 0.000 | 0.000 | 0.006 | 0.484 | 0.004 | 0.000 | 0.103 | 0.000 | 0.009 | 0.172 | 0.004 |
| **LKM_CDP** | 0.001 | 0.000 | 0.110 | 0.001 | 0.000 | 0.129 | 0.023 | 0.025 | 0.005 | 0.001 | 0.418 | 0.001 | 0.000 | 0.110 | 0.001 | 0.000 | 0.174 |

Table A2.3 continued. For the Au Sable River random forest analysis, the proportion of assignments to the correct model are represented on the diagonal of the table; whereas, the out-of-bag error rates represent the proportion of datasets misassigned to alternative models are represented in the remainder of the table. The first column and row (in bold) display abbreviated model names that represent the source of the introduction (name to the left of the underscore), and the first location(s) that were founded within the system (to the right of the underscore). CDP+FCP represents introductions into both Cook Dam Pond and Five Channels Pond at the same time. ‘Local’ models represent upstream stepping-stone models via bait-bucket movements. LocalSAB represents Round Gobies collected as bait in Saginaw Bay that were brought into the system and then moved upstream. Each Time event represent branching events on the trees – i.e. new founding events. Abbreviations for locations are as follows: LKM – Lake Michigan, STC – Lake St. Clair, LKE – Lake Erie, SAB – Saginaw Bay, ALP – Alpena, FDP – Foote Dam Pond, CDP – Cook Dam Pond, FCP – Five Channels Pond. See Table A1.3 (Appendix 1) for description of models.

|  | **ALP_FCP** | **ALP_CDP+FCP** | **ALP_CDP** | **LKE_FCP** | **LKE_CDP+FCP** | **LKE_CDP** | **LocalALP** | **LocalSAB** | **LKM_FCP** | **LKM_CDP+FCP** | **LKM_CDP** | **SAB_FCP** | **SAB_CDP+FCP** | **SAB_CDP** | **STC_FCP** | **STC_CDP+FCP** | **STC_CDP** |
| --- | --- | --- | --- | --- | --- | --- | --- | --- | --- | --- | --- | --- | --- | --- | --- | --- | --- |
| **SAB_FCP** | 0.084 | 0.000 | 0.001 | 0.068 | 0.000 | 0.001 | 0.001 | 0.003 | 0.076 | 0.000 | 0.002 | 0.685 | 0.001 | 0.015 | 0.062 | 0.000 | 0.001 |
| **SAB_CDP+FCP** | 0.000 | 0.065 | 0.000 | 0.000 | 0.053 | 0.000 | 0.000 | 0.000 | 0.000 | 0.054 | 0.000 | 0.007 | 0.753 | 0.005 | 0.000 | 0.063 | 0.000 |
| **SAB_CDP** | 0.001 | 0.000 | 0.077 | 0.000 | 0.000 | 0.064 | 0.011 | 0.052 | 0.001 | 0.000 | 0.070 | 0.007 | 0.001 | 0.654 | 0.000 | 0.000 | 0.061 |
| **STC_FCP** | 0.139 | 0.000 | 0.003 | 0.108 | 0.000 | 0.002 | 0.001 | 0.001 | 0.258 | 0.004 | 0.010 | 0.129 | 0.000 | 0.002 | 0.336 | 0.001 | 0.005 |
| **STC_CDP+FCP** | 0.000 | 0.111 | 0.000 | 0.000 | 0.088 | 0.000 | 0.000 | 0.000 | 0.000 | 0.152 | 0.000 | 0.000 | 0.108 | 0.000 | 0.001 | 0.538 | 0.001 |
| **STC_CDP** | 0.001 | 0.000 | 0.129 | 0.001 | 0.000 | 0.101 | 0.024 | 0.024 | 0.004 | 0.001 | 0.249 | 0.001 | 0.000 | 0.122 | 0.002 | 0.001 | 0.340 |

Table A2.4. For the Cheboygan River random forest analysis, the proportion of assignments to the correct model are represented on the diagonal of the table; whereas, the out-of-bag error rates represent the proportion of datasets misassigned to alternative models are represented in the remainder of the table. The first column and row (in bold) display abbreviated model names that represent the source of the introduction (name to the left of the underscore), and the first location(s) that were founded within the system (to the right of the underscore). BTL+MLL represents introductions into both Burt Lake and Mullett Lake at the same time. ‘Local’ models represent upstream stepping-stone models via bait-bucket movements. LocalEAST represents Round Gobies collected as bait in the Cheboygan River that were brought into the system from east to west. Abbreviations for locations are as follows: LKM – Lake Michigan, STC – Lake St. Clair, LKE – Lake Erie, SAB – Saginaw Bay, ALP – Alpena, GTB – Grand Traverse Bay, MLL – Mullett Lake, and BTL – Burt Lake. See Table A1.4 (Appendix 1) for description of models.

|  | **ALP_BTL** | **ALP_BTL+MLL** | **ALP_MLL** | **LKE_BTL** | **LKE_BTL+MLL** | **LKE_MLL** | **GTB_BTL** | **BTB_BTL+MLL** | **GTB_MLL** | **LocalBOTH** | **LocalEAST** | **LocalWEST** | **LKM_BTL** | **LKM_BTL+MLL** | **LKM_MLL** | **SAB_BTL** | **SAB_BTL+MLL** | **SAB_MLL** | **STC_BTL** | **STC_BTL+MLL** | **STC_MLL** |
| --- | --- | --- | --- | --- | --- | --- | --- | --- | --- | --- | --- | --- | --- | --- | --- | --- | --- | --- | --- | --- | --- |
| **ALP_BTL** | 0.722 | 0.006 | 0.053 | 0.038 | 0.000 | 0.003 | 0.019 | 0.000 | 0.002 | 0.000 | 0.014 | 0.014 | 0.026 | 0.000 | 0.002 | 0.041 | 0.000 | 0.003 | 0.054 | 0.000 | 0.004 |
| **ALP_BTL+MLL** | 0.017 | 0.771 | 0.019 | 0.000 | 0.032 | 0.000 | 0.000 | 0.016 | 0.000 | 0.028 | 0.003 | 0.000 | 0.000 | 0.022 | 0.000 | 0.000 | 0.036 | 0.000 | 0.001 | 0.054 | 0.001 |
| **ALP_MLL** | 0.054 | 0.003 | 0.644 | 0.003 | 0.000 | 0.033 | 0.001 | 0.000 | 0.025 | 0.000 | 0.114 | 0.001 | 0.002 | 0.000 | 0.026 | 0.003 | 0.000 | 0.036 | 0.004 | 0.000 | 0.050 |
| **LKE_BTL** | 0.048 | 0.000 | 0.003 | 0.704 | 0.006 | 0.058 | 0.022 | 0.000 | 0.002 | 0.000 | 0.003 | 0.015 | 0.033 | 0.000 | 0.003 | 0.042 | 0.000 | 0.003 | 0.053 | 0.000 | 0.003 |
| **LKE_BTL+MLL** | 0.000 | 0.037 | 0.000 | 0.016 | 0.773 | 0.019 | 0.000 | 0.020 | 0.000 | 0.015 | 0.000 | 0.000 | 0.000 | 0.028 | 0.000 | 0.000 | 0.037 | 0.000 | 0.001 | 0.052 | 0.001 |
| **LKE_MLL** | 0.003 | 0.000 | 0.036 | 0.057 | 0.004 | 0.699 | 0.002 | 0.000 | 0.033 | 0.000 | 0.032 | 0.001 | 0.003 | 0.000 | 0.036 | 0.003 | 0.000 | 0.039 | 0.004 | 0.000 | 0.048 |
| **GTB_BTL** | 0.005 | 0.000 | 0.000 | 0.006 | 0.000 | 0.000 | 0.634 | 0.007 | 0.063 | 0.000 | 0.000 | 0.246 | 0.025 | 0.000 | 0.002 | 0.005 | 0.000 | 0.000 | 0.007 | 0.000 | 0.000 |
| **BTB_BTL+MLL** | 0.000 | 0.003 | 0.000 | 0.000 | 0.004 | 0.000 | 0.021 | 0.885 | 0.033 | 0.022 | 0.000 | 0.006 | 0.000 | 0.017 | 0.000 | 0.000 | 0.004 | 0.000 | 0.000 | 0.004 | 0.000 |
| **GTB_MLL** | 0.000 | 0.000 | 0.005 | 0.000 | 0.000 | 0.005 | 0.053 | 0.004 | 0.866 | 0.000 | 0.006 | 0.028 | 0.002 | 0.000 | 0.020 | 0.000 | 0.000 | 0.005 | 0.000 | 0.000 | 0.006 |
| **LocalBOTH** | 0.000 | 0.004 | 0.000 | 0.000 | 0.003 | 0.000 | 0.000 | 0.014 | 0.000 | 0.964 | 0.001 | 0.001 | 0.000 | 0.005 | 0.000 | 0.000 | 0.003 | 0.000 | 0.000 | 0.004 | 0.000 |
| **LocalEAST** | 0.006 | 0.000 | 0.026 | 0.001 | 0.000 | 0.008 | 0.001 | 0.000 | 0.011 | 0.000 | 0.920 | 0.001 | 0.001 | 0.000 | 0.007 | 0.001 | 0.000 | 0.008 | 0.001 | 0.000 | 0.010 |
| **LocalWEST** | 0.003 | 0.000 | 0.000 | 0.003 | 0.000 | 0.000 | 0.098 | 0.002 | 0.026 | 0.000 | 0.000 | 0.851 | 0.009 | 0.000 | 0.001 | 0.003 | 0.000 | 0.000 | 0.004 | 0.000 | 0.000 |

Table A2.4 continued. For the Cheboygan River random forest analysis, the proportion of assignments to the correct model are represented on the diagonal of the table; whereas, the out-of-bag error rates represent the proportion of datasets misassigned to alternative models are represented in the remainder of the table. The first column and row (in bold) display abbreviated model names that represent the source of the introduction (name to the left of the underscore), and the first location(s) that were founded within the system (to the right of the underscore). BTL+MLL represents introductions into both Burt Lake and Mullett Lake at the same time. ‘Local’ models represent upstream stepping-stone models via bait-bucket movements. LocalEAST represents Round Gobies collected as bait in the Cheboygan River that were brought into the system from east to west. Abbreviations for locations are as follows: LKM – Lake Michigan, STC – Lake St. Clair, LKE – Lake Erie, SAB – Saginaw Bay, ALP – Alpena, GTB – Grand Traverse Bay, MLL – Mullett Lake, and BTL – Burt Lake. See Table A1.4 (Appendix 1) for description of models.

|  | **ALP_BTL** | **ALP_BTL+MLL** | **ALP_MLL** | **LKE_BTL** | **LKE_BTL+MLL** | **LKE_MLL** | **GTB_BTL** | **BTB_BTL+MLL** | **GTB_MLL** | **LocalBOTH** | **LocalEAST** | **LocalWEST** | **LKM_BTL** | **LKM_BTL+MLL** | **LKM_MLL** | **SAB_BTL** | **SAB_BTL+MLL** | **SAB_MLL** | **STC_BTL** | **STC_BTL+MLL** | **STC_MLL** |
| --- | --- | --- | --- | --- | --- | --- | --- | --- | --- | --- | --- | --- | --- | --- | --- | --- | --- | --- | --- | --- | --- |
| **LKM_BTL** | 0.059 | 0.000 | 0.004 | 0.065 | 0.000 | 0.005 | 0.083 | 0.001 | 0.009 | 0.000 | 0.003 | 0.034 | 0.522 | 0.003 | 0.044 | 0.061 | 0.000 | 0.004 | 0.095 | 0.001 | 0.006 |
| **LKM_BTL+MLL** | 0.001 | 0.050 | 0.001 | 0.001 | 0.061 | 0.001 | 0.001 | 0.090 | 0.002 | 0.023 | 0.000 | 0.000 | 0.015 | 0.565 | 0.018 | 0.001 | 0.058 | 0.000 | 0.003 | 0.107 | 0.003 |
| **LKM_MLL** | 0.004 | 0.000 | 0.046 | 0.005 | 0.000 | 0.060 | 0.007 | 0.000 | 0.123 | 0.000 | 0.027 | 0.003 | 0.043 | 0.002 | 0.525 | 0.005 | 0.000 | 0.055 | 0.006 | 0.000 | 0.087 |
| **SAB_BTL** | 0.074 | 0.000 | 0.005 | 0.060 | 0.000 | 0.005 | 0.022 | 0.000 | 0.002 | 0.000 | 0.004 | 0.016 | 0.040 | 0.000 | 0.003 | 0.632 | 0.005 | 0.051 | 0.073 | 0.001 | 0.005 |
| **SAB_BTL+MLL** | 0.001 | 0.060 | 0.001 | 0.000 | 0.054 | 0.000 | 0.000 | 0.020 | 0.000 | 0.018 | 0.000 | 0.000 | 0.000 | 0.036 | 0.000 | 0.015 | 0.696 | 0.019 | 0.001 | 0.076 | 0.001 |
| **SAB_MLL** | 0.005 | 0.000 | 0.057 | 0.005 | 0.000 | 0.056 | 0.002 | 0.000 | 0.034 | 0.000 | 0.039 | 0.001 | 0.003 | 0.000 | 0.044 | 0.051 | 0.003 | 0.627 | 0.005 | 0.000 | 0.068 |
| **STC_BTL** | 0.111 | 0.001 | 0.008 | 0.091 | 0.000 | 0.007 | 0.023 | 0.000 | 0.002 | 0.000 | 0.005 | 0.016 | 0.073 | 0.000 | 0.007 | 0.105 | 0.001 | 0.008 | 0.498 | 0.006 | 0.037 |
| **STC_BTL+MLL** | 0.001 | 0.089 | 0.001 | 0.001 | 0.082 | 0.001 | 0.000 | 0.020 | 0.000 | 0.017 | 0.000 | 0.000 | 0.000 | 0.066 | 0.001 | 0.001 | 0.095 | 0.001 | 0.008 | 0.605 | 0.012 |
| **STC_MLL** | 0.008 | 0.000 | 0.089 | 0.007 | 0.000 | 0.087 | 0.002 | 0.000 | 0.035 | 0.000 | 0.042 | 0.001 | 0.006 | 0.000 | 0.081 | 0.009 | 0.000 | 0.100 | 0.039 | 0.004 | 0.489 |


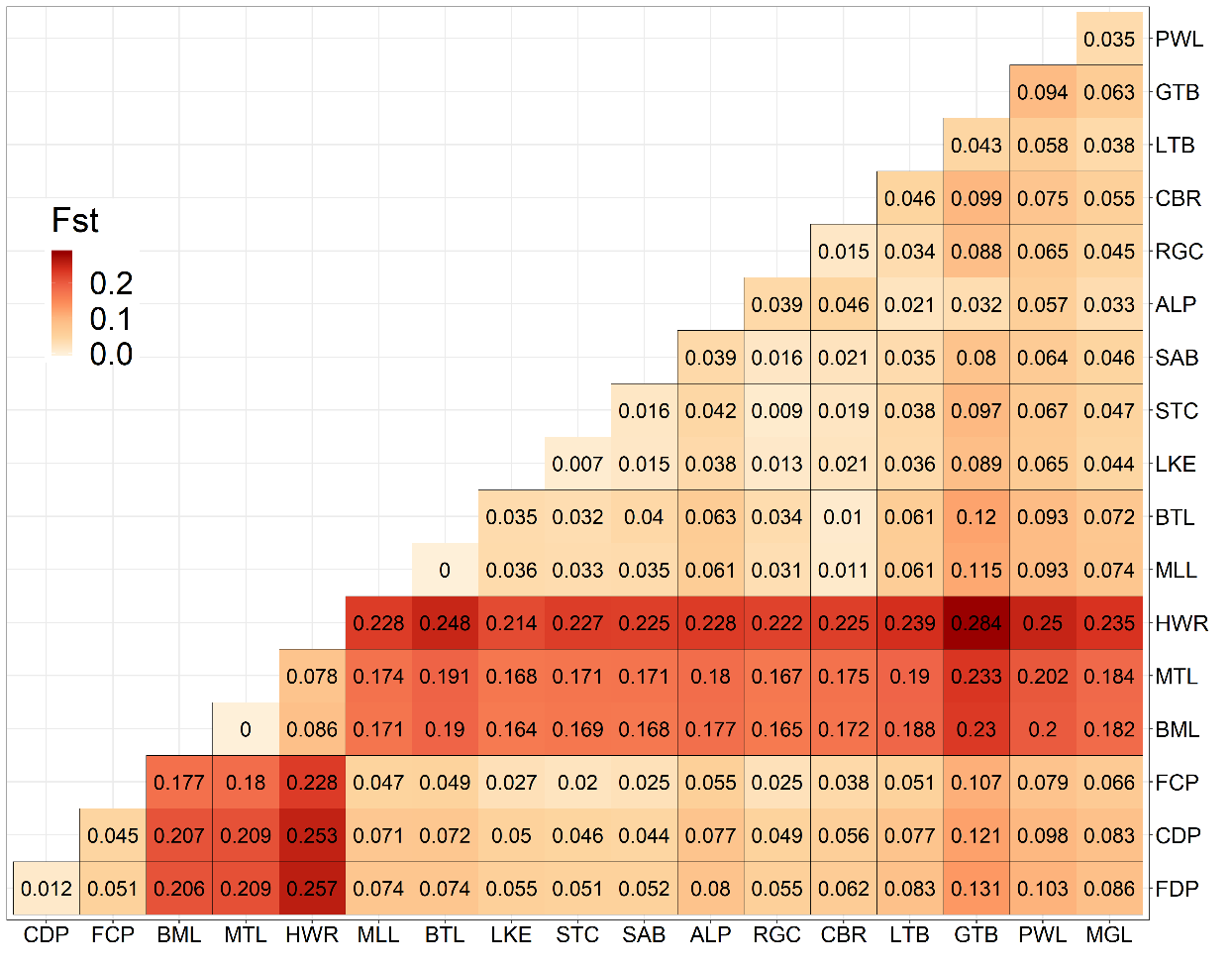


Figure A2.1. Pairwise F_ST_ values for inland and Great Lakes sampling location. Light to dark red colors indicate low to high F_ST_ estimates. Abbreviations for sampling locations are as follows: STC – Lake St. Clair, LKE – Lake Erie, SAB – Saginaw Bay, ALP – Alpena, MGL – Muskegon Lake, RGC – Roger City, CBR – Cheboygan River, LTB – Little Traverse Bay, GTB – Grand Traverse Bay, GTB – Grand Traverse Bay, LTB – Little Traverse Bay, MLL – Mullett Lake, BTL – Burt Lake, FDP – Foote Dam Pond, CDP – Cook Dam Pond, FCP – Five Channels Pond, BML – Below Mott Lake, MTL – Mott Lake, HWR – Holloway Reservoir.


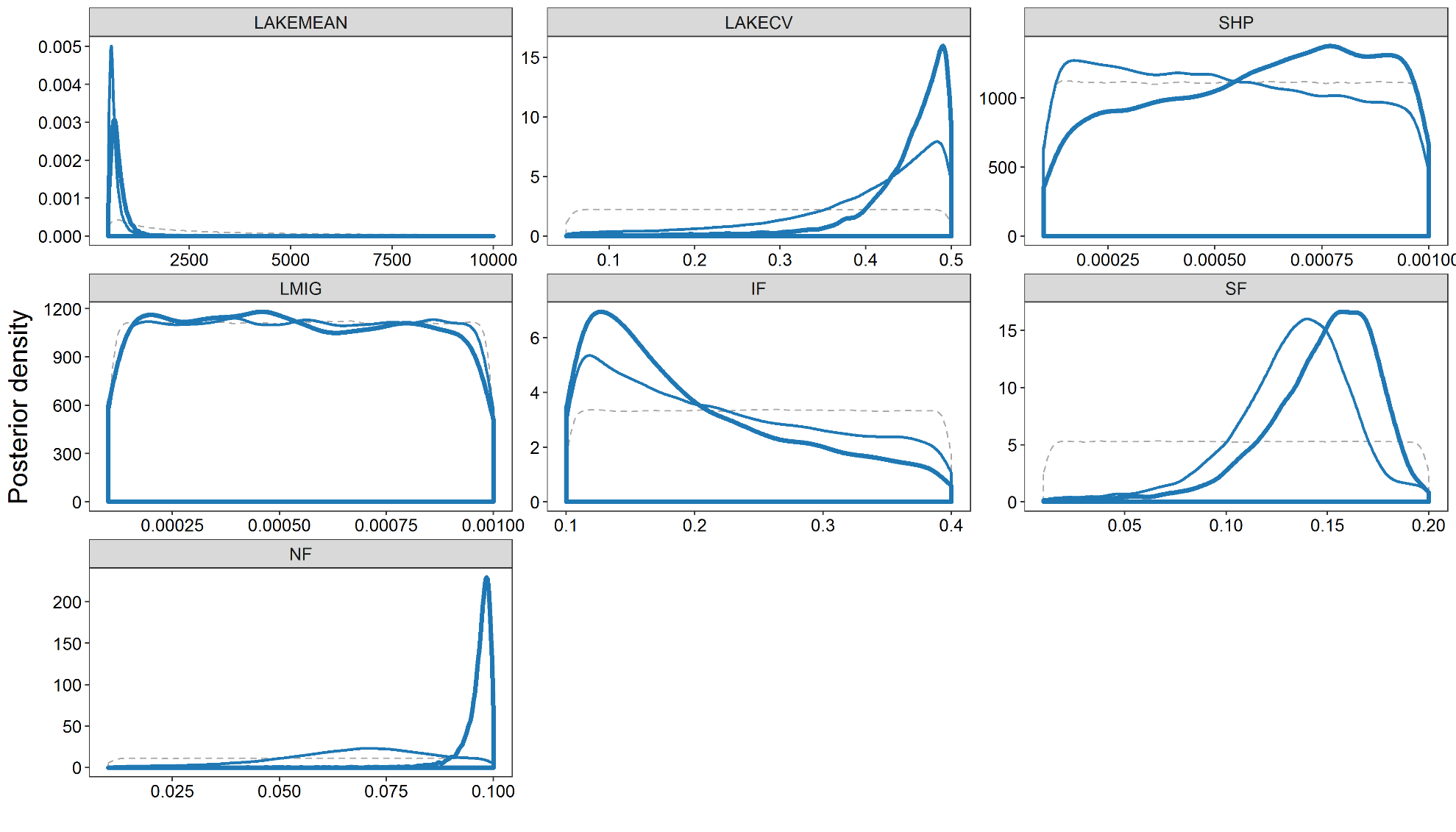


Figure A2.2. Prior (dotted lines) and posterior probability densities for demographic parameters estimated in the Lower Peninsula analysis. Thin and thick lines represent posterior estimates at 0.01 and 0.005 tolerances for the CBR model in the Lower Peninsula analysis. See Table A1.1 (Appendix 1) for description of the model.


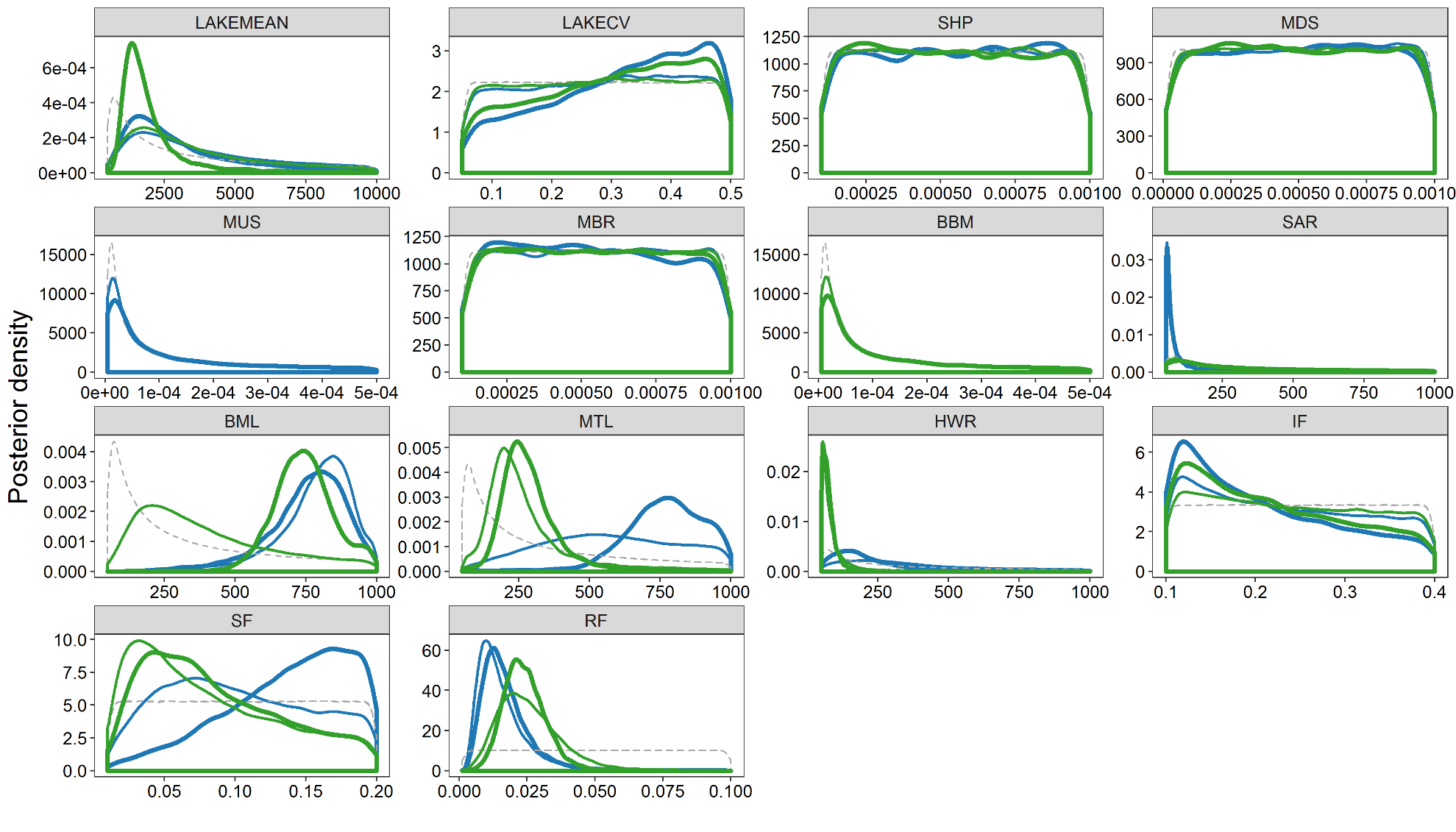


Figure A2.3. Prior (dotted lines) and posterior probability densities for demographic parameters estimated in the Flint analysis. Thin and thick lines represent posterior estimates at 0.05 and 0.01 tolerances for each supported model. LocalSAB (dark blue) and SAB_HWR (dark green) models in the Flint River analysis are represented. See Table A1.2 (Appendix 1) for description of the models.


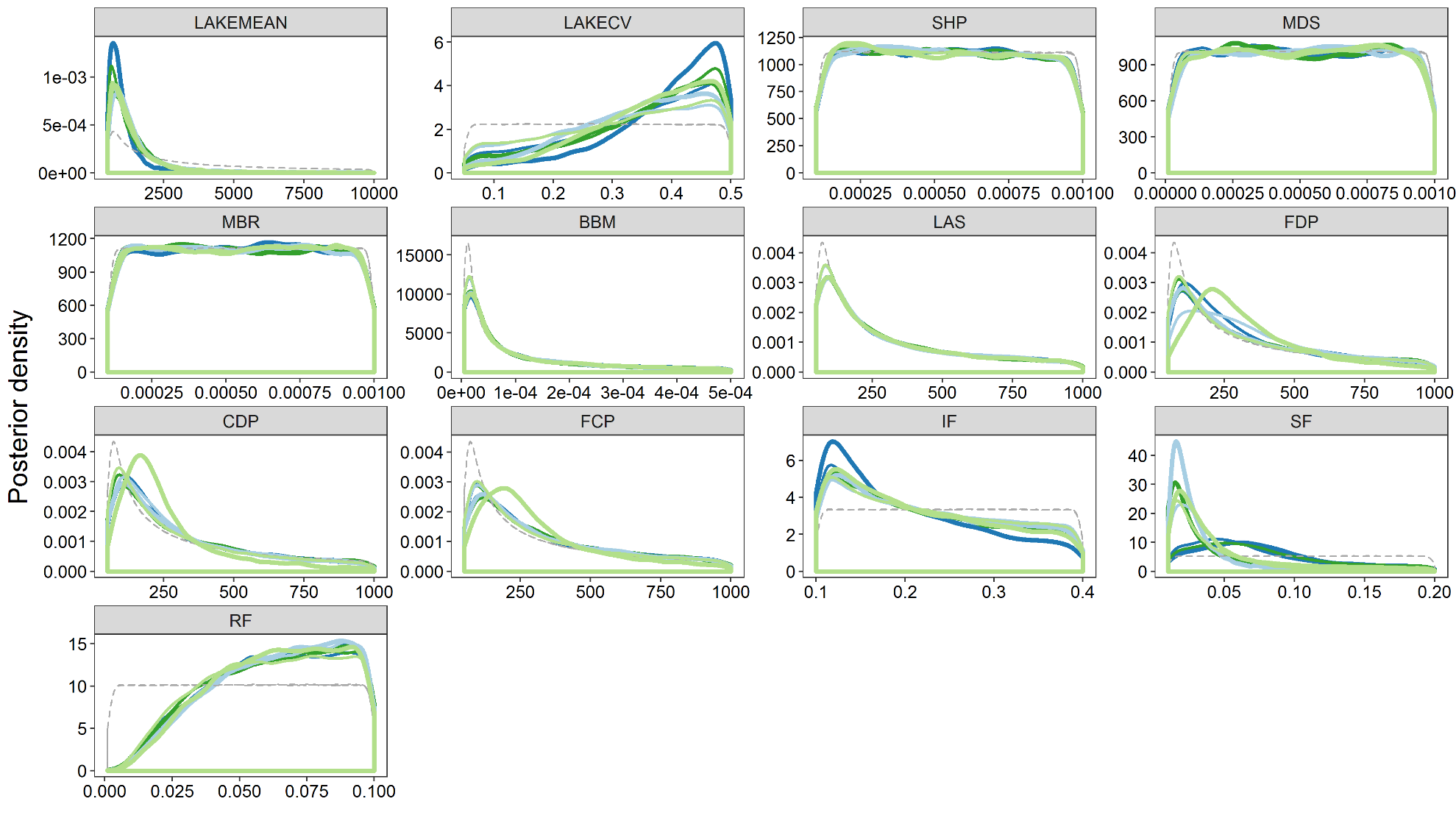


Figure A2.4. Prior (dotted lines) and posterior probability densities for demographic parameters estimated in the Au Sable analysis. Thin and thick lines represent posterior estimates at 0.05 and 0.01 tolerances for each supported model. LKE_CDP+FCP (dark blue), LKM_CDP+FCP (dark green), SAB_CDP+FCP (light blue), and STC_CDP+FCP (light green) models in the Au Sable River analysis are represented from light to dark grey lines, respectively. See Table A1.3 (Appendix 1) for description of the models.


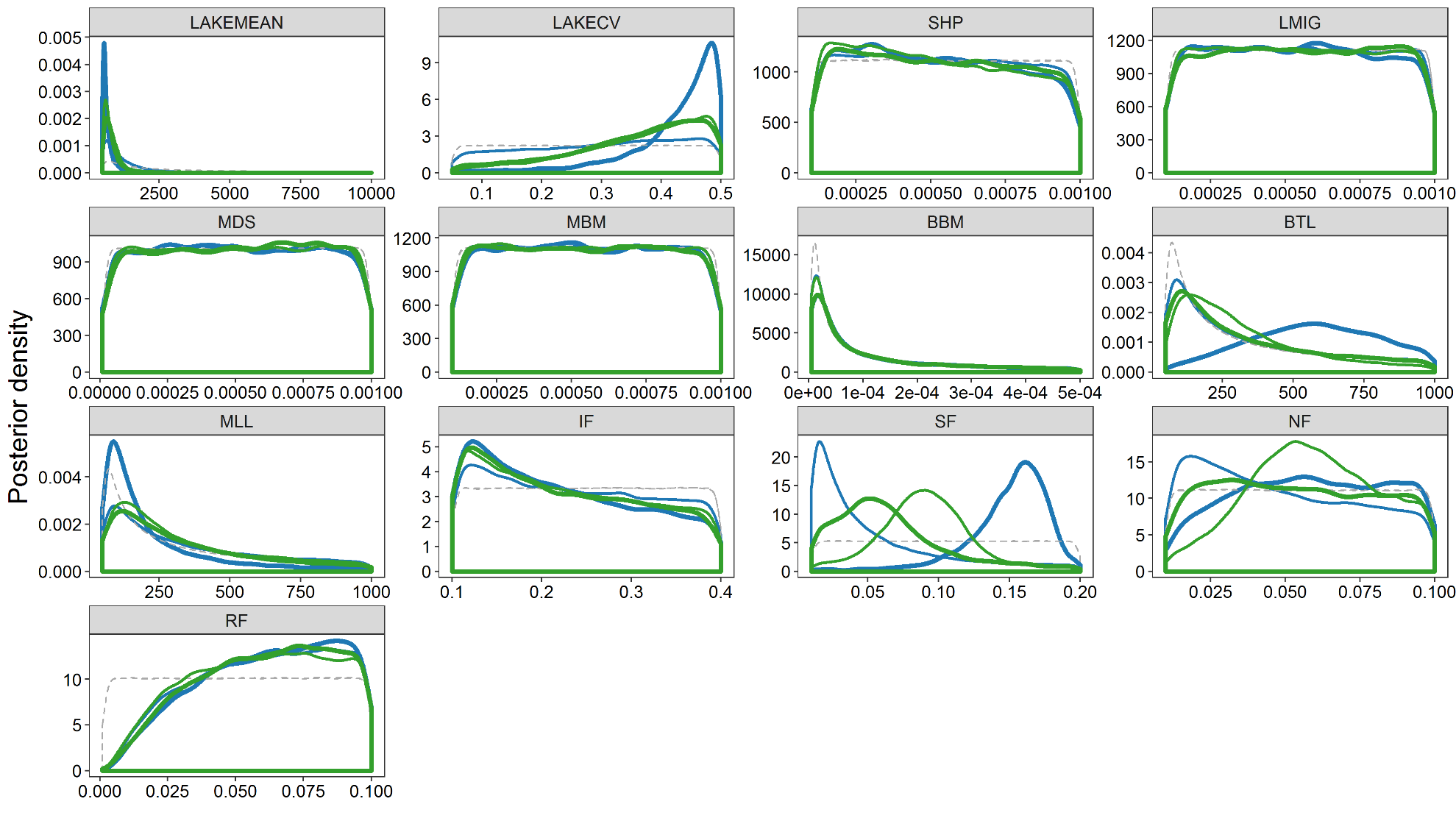


Figure A2.5. Prior (dotted lines) and posterior probability densities for demographic parameters estimated in the Cheboygan River analysis. Thin and thick lines represent posterior estimates at 0.05 and 0.01 tolerances for each supported model. LocalEAST (dark blue) and SAB_MLL (dark green) models in the Cheboygan River analysis are represented. See Table A1.4 (Appendix 1) for description of the models.


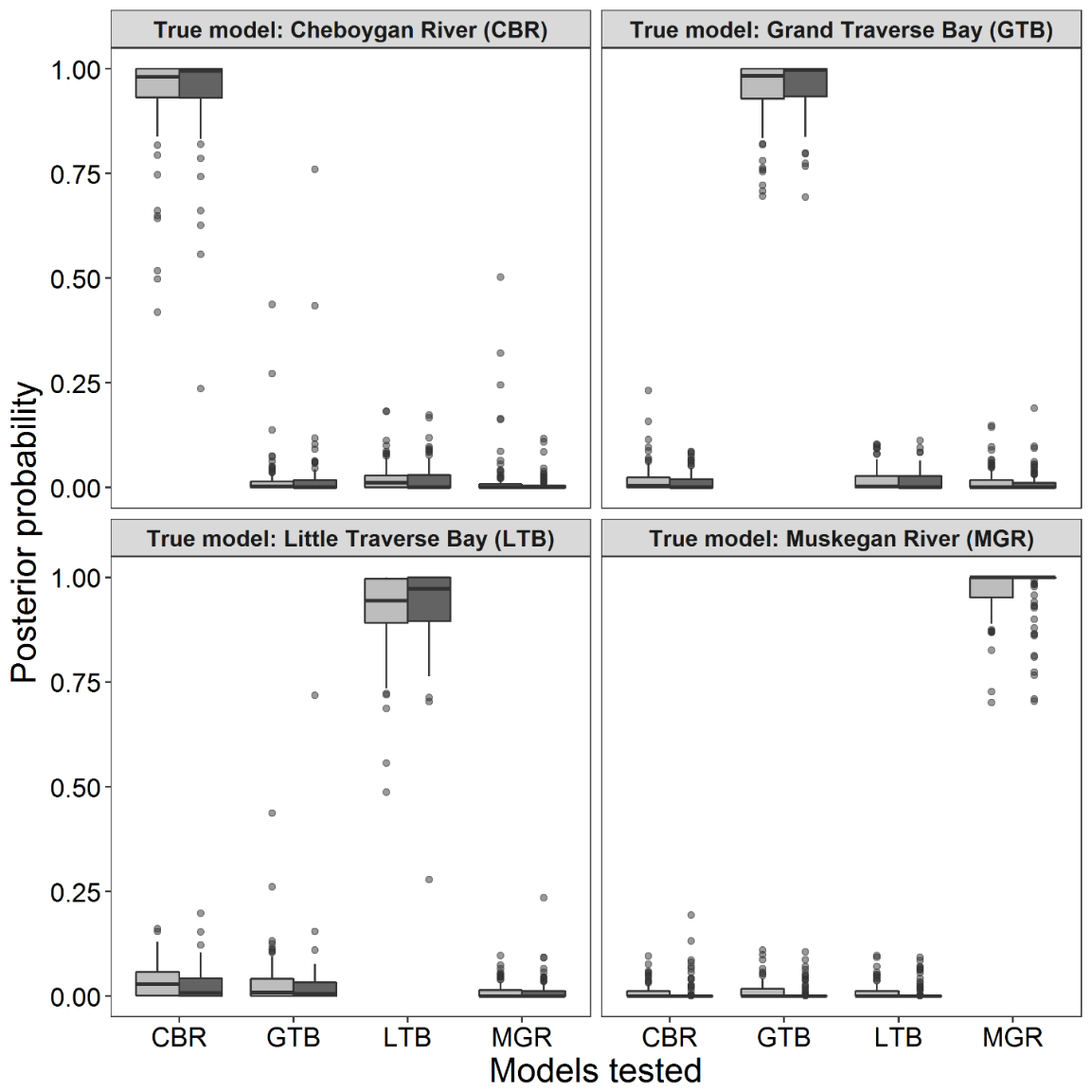


Figure A2.6. Boxplots depicting 100 leave-one-out cross-validations for neural network analysis at tolerances 0.01 (Light grey) and 0.005 (dark grey) for the Lower Peninsula analysis. See Table A1.1 (Appendix 1) for description of the model.


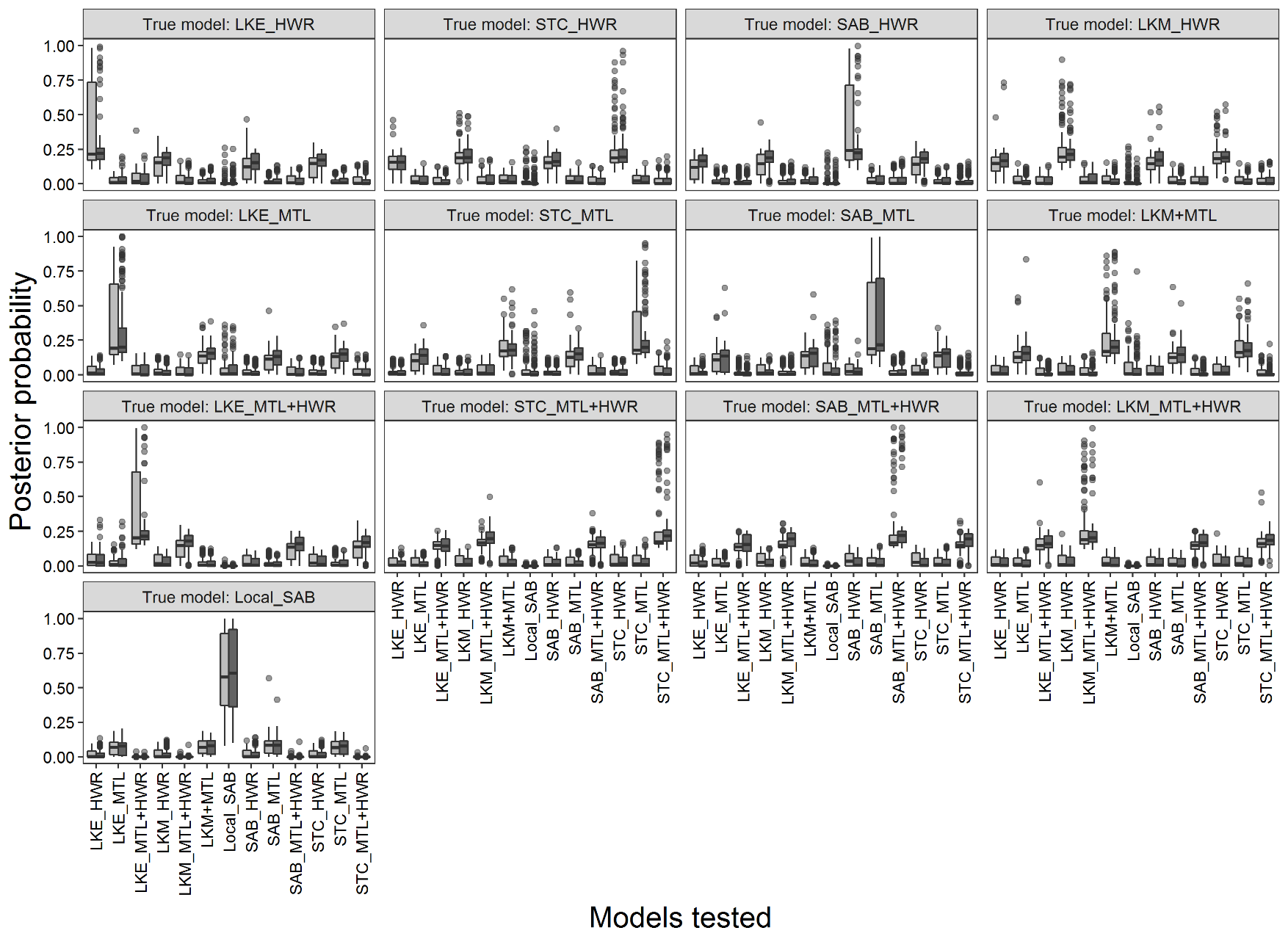


Figure A2.7. Boxplots depicting 100 leave-one-out cross-validations for neural network analysis at tolerances 0.005 (Light grey) and 0.001 (dark grey) for the Flint River analysis. See Table A1.2 (Appendix 1) for description of the models.


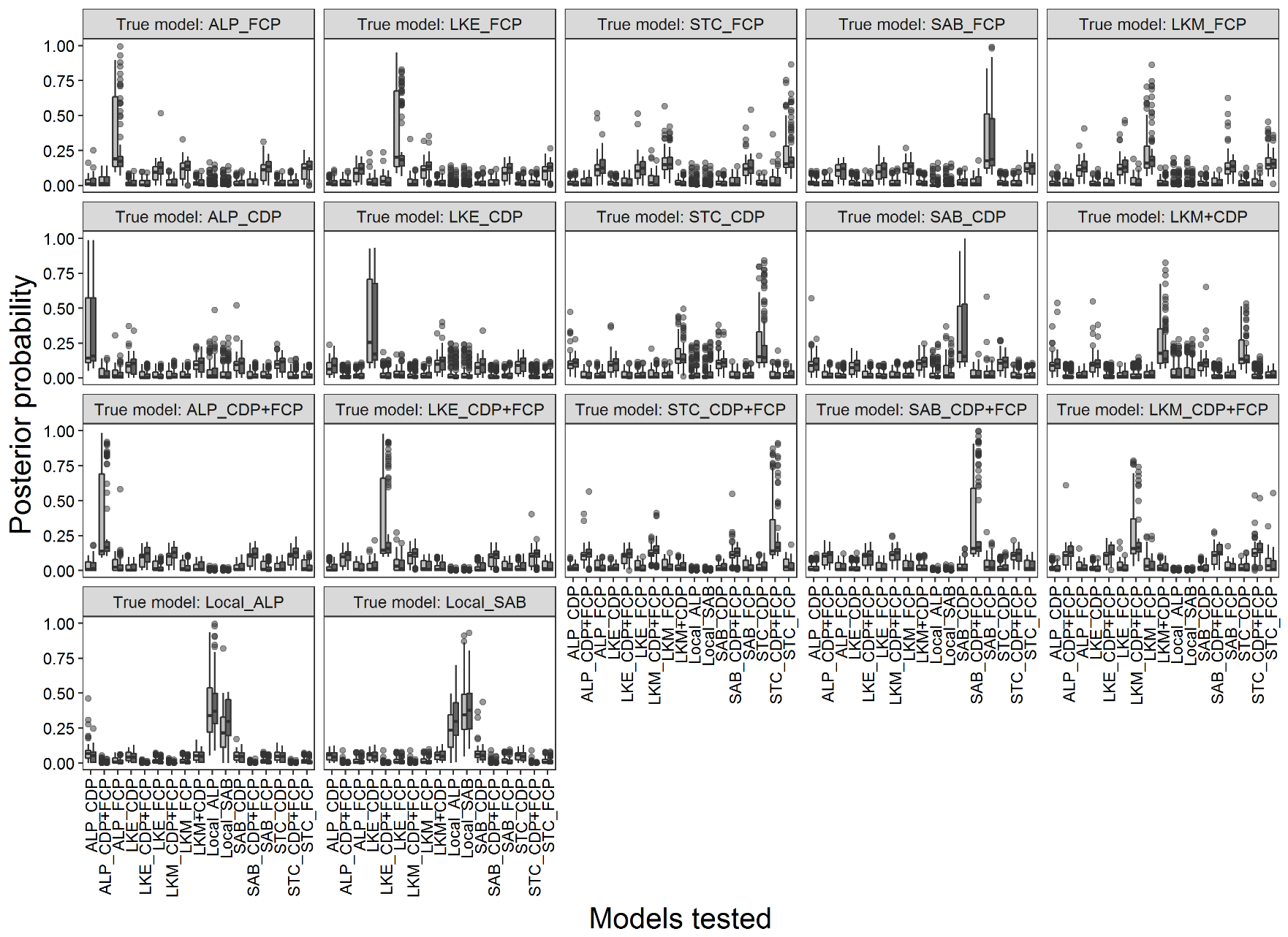


Figure A2.8. Boxplots depicting 100 leave-one-out cross-validations for neural network analysis at tolerances 0.005 (Light grey) and 0.001 (dark grey) for the Au Sable River analysis. See Table A1.3 (Appendix 1) for description of the models.


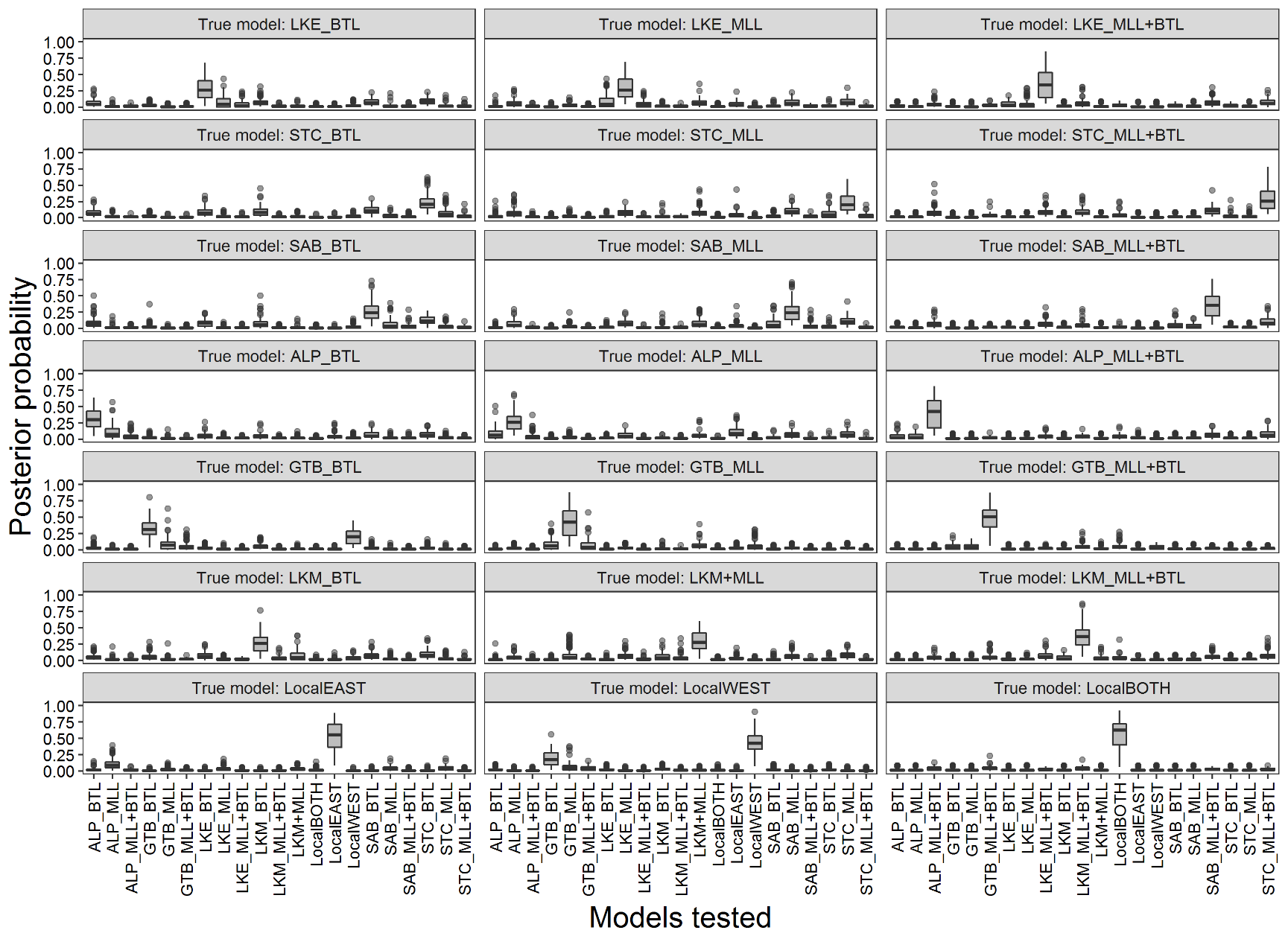


Figure A2.9. Boxplots depicting 100 leave-one-out cross-validations for neural network analysis at tolerances 0.005 (Light grey) and 0.001 (dark grey) for the Cheboygan River analysis. See Table A1.4 (Appendix 1) for description of the models.


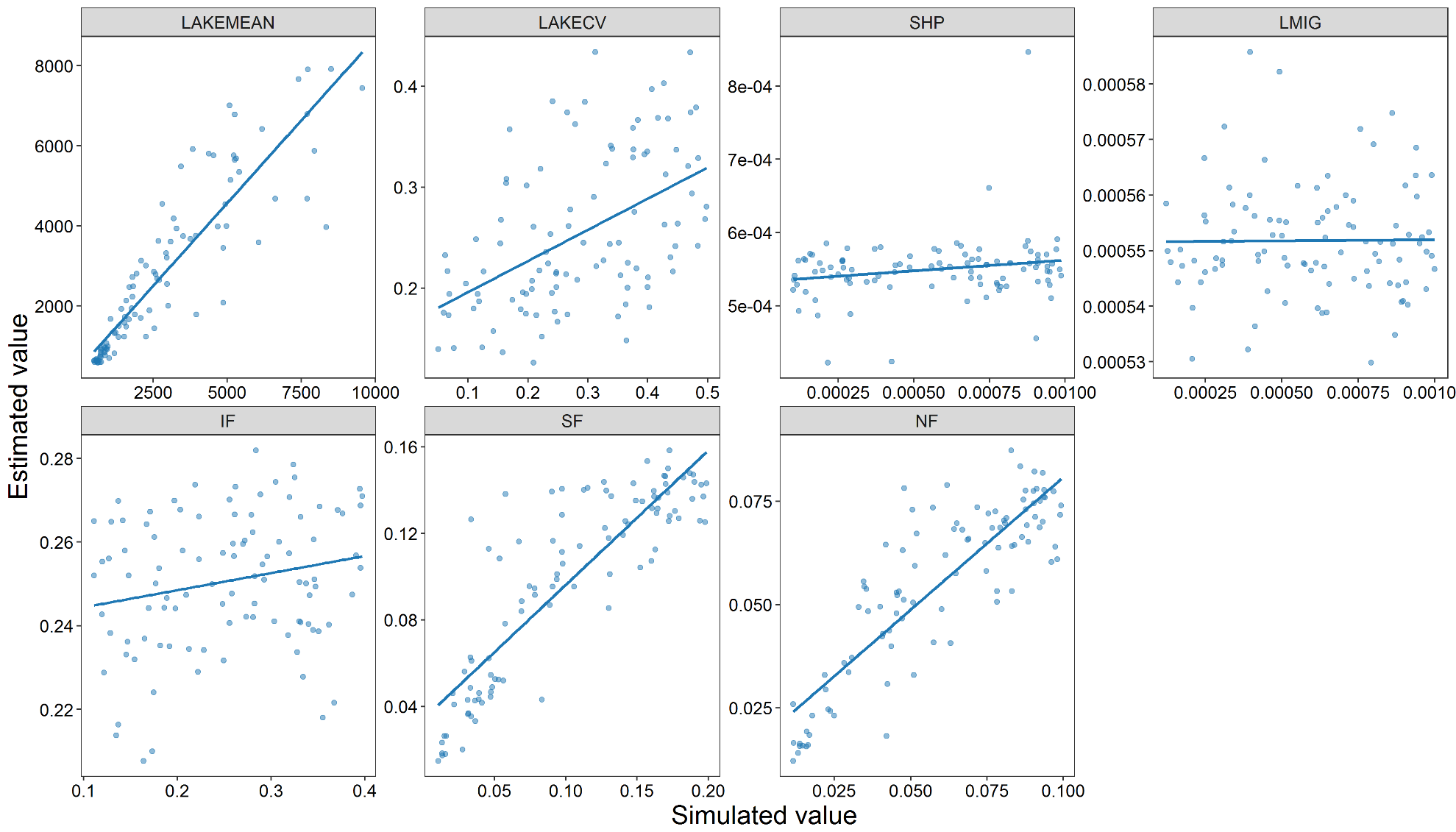


Figure A2.10. Relationships between simulated (known) and estimated parameters based on 100 leave-one-out cross-validations for neural network analysis at a tolerance of 0.01 (blue) for the CBR model in the Lower Peninsula analysis. See Table A1.1 (Appendix 1) for description of the model.


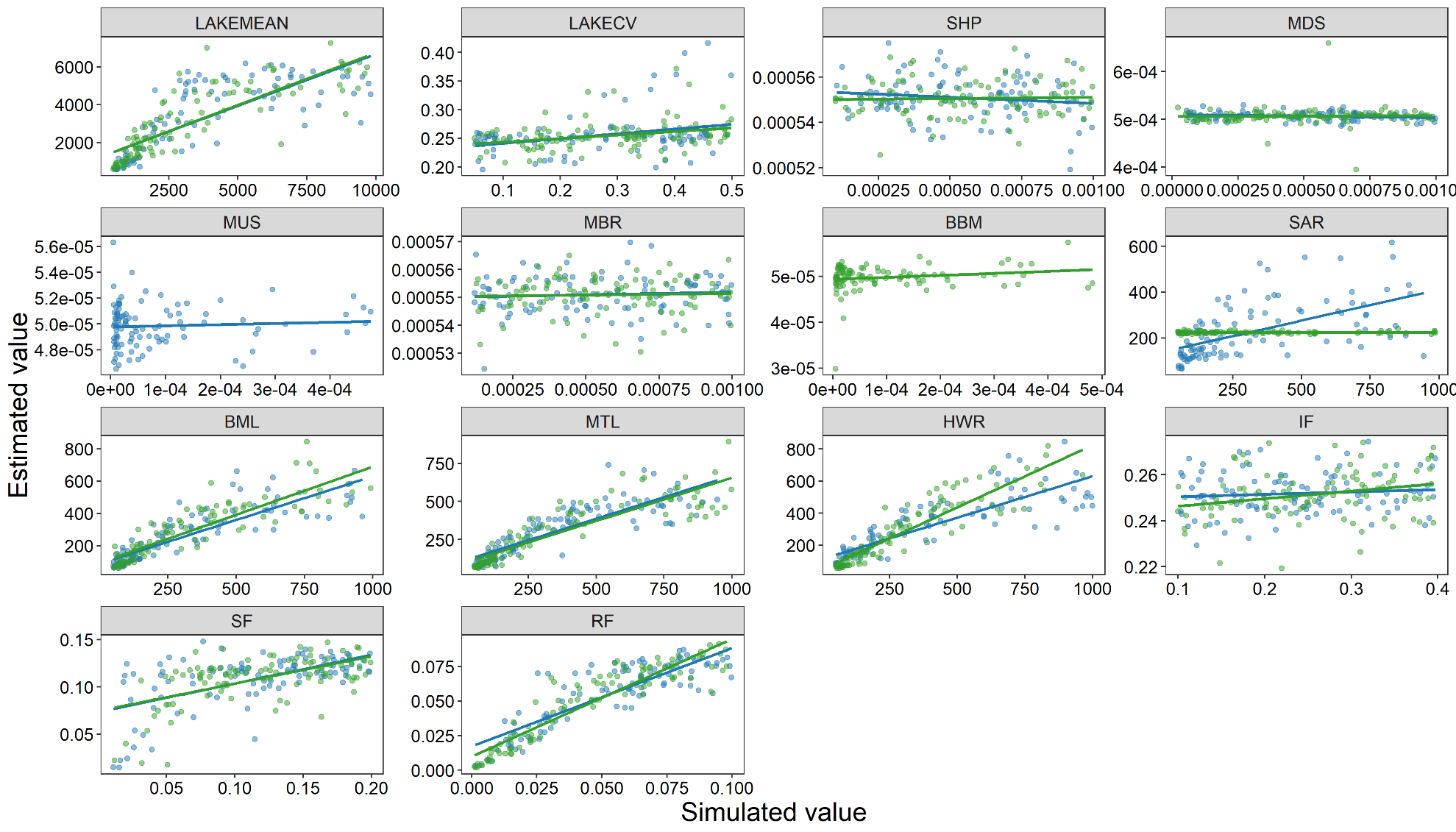


Figure A2.11. Relationships between simulated (known) and estimated parameters based on 100 leave-one-out cross-validations for neural network analysis at a tolerance of 0.01 (dark grey). LocalSAB (dark blue) and SAB_HWR (dark green) models in the Flint River analysis are represented. See Table A1.2 (Appendix 1) for description of the models.


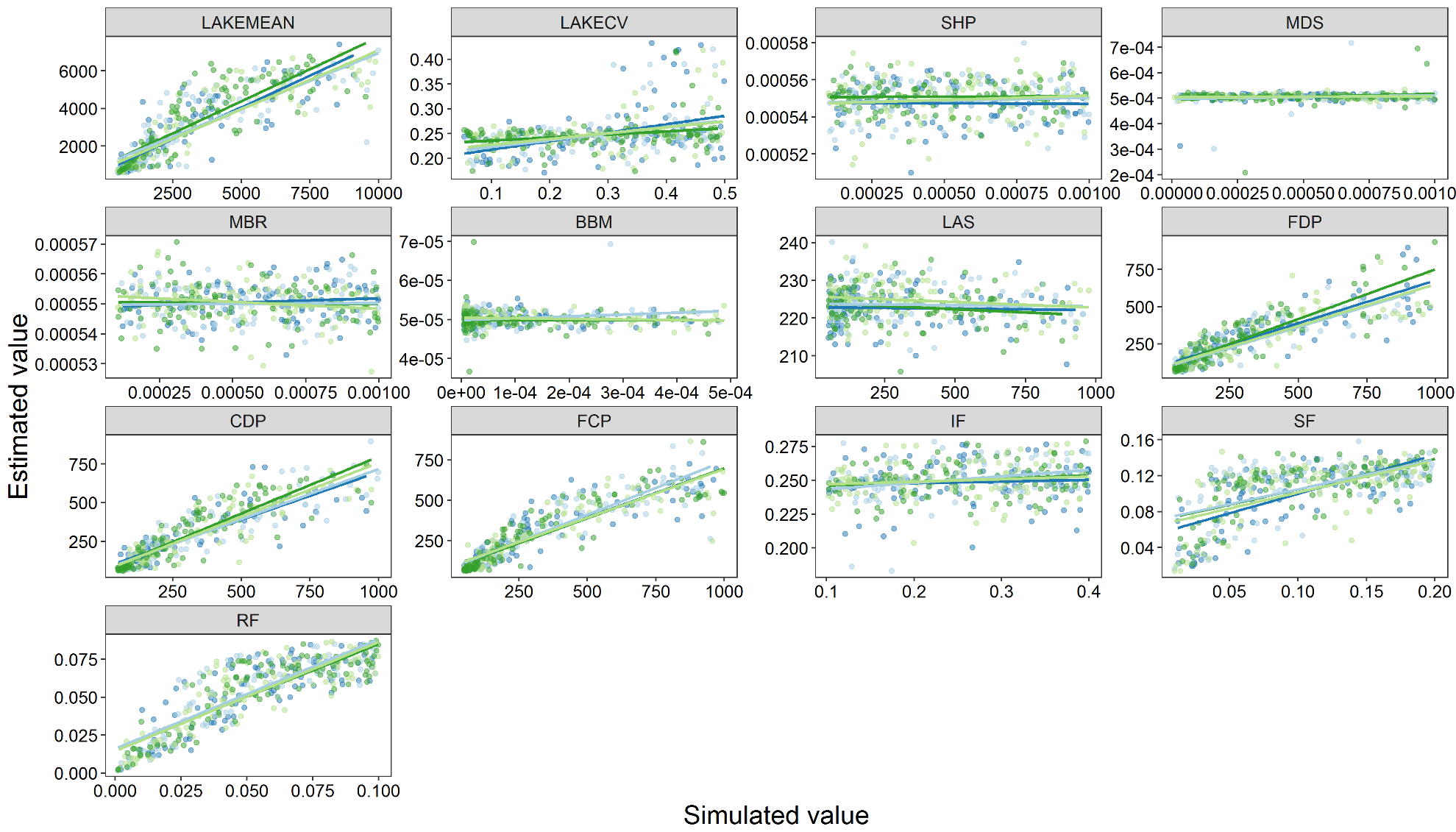


Figure A2.12. Relationships between simulated (known) and estimated parameters based on 100 leave-one-out cross-validations for neural network analysis at a tolerance of 0.01 (dark grey). LKE_CDP+FCP (dark blue), LKM_CDP+FCP (dark green), SAB_CDP+FCP (light blue), and STC_CDP+FCP (light green) models in the Au Sable River analysis are represented from light to dark grey lines, respectively. See Table A1.3 (Appendix 1) for description of the models.


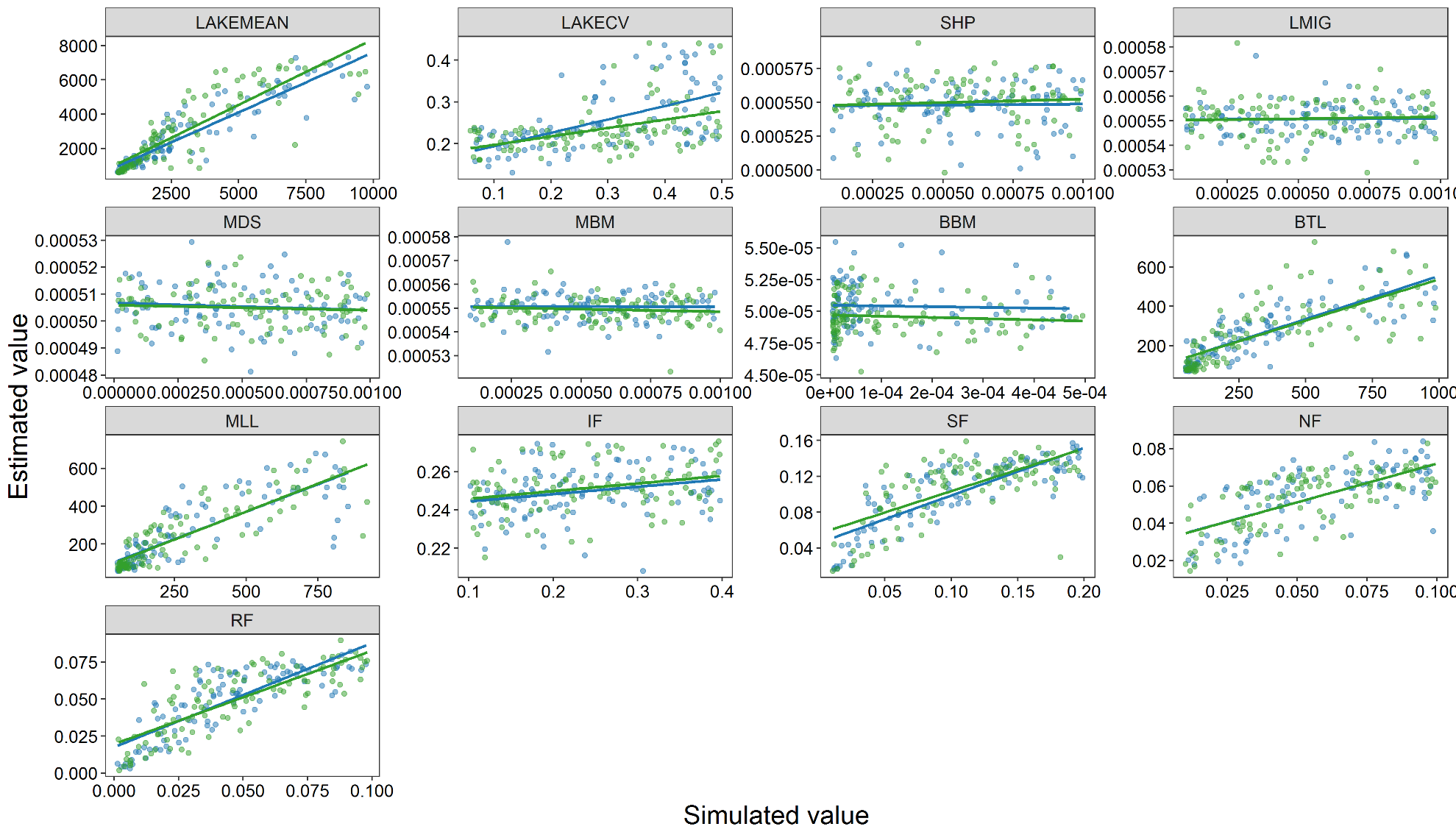


Figure A2.13. Relationships between simulated (known) and estimated parameters based on 100 leave-one-out cross-validations for neural network analysis at a tolerance of 0.01 (dark grey). LocalEAST and SAB_MLL models in the Cheboygan River analysis are represented by light and dark grey lines/points, respectively. See Table A1.4 (Appendix 1) for description of the models.
